# Supplementary material for: A protocol for an economic evaluation of a polypill in patients with established or at high risk of cardiovascular disease in a UK NHS setting: RUPEE (NHS) study
Source: BMJ Open. 2018 Mar 14;8(3):e013063. doi: 10.1136/bmjopen-2016-013063 (PMC5857692; doi:10.1136/bmjopen-2016-013063)
Supplement: Supplementary data [file bmjopen-2016-013063supp001.pdf]

Supplementary appendix

Section 1- Literature review

The purpose of the literature review was to identify the appropriate analytical framework to represent the decision problem. The literature review also aimed to inform the underlying model structure: disease and care pathway.

A general search of the literature identified a known review of coronary heart disease policy models by Unal et al. (2006).(1) This review was updated and expanded in 2008 by Capewell et al. to include stroke models. (2) A search carried out in Medline found no further systematic reviews of coronary heart disease or cardiovascular disease models published since 2008. The review by Capewell et al. (2008) identified seven notable CHD models (six of these had been identified in the previous review by Unal et al), nine stroke models and several models that were currently in development at the time of publication. The notable models and models in development were reviewed. Additionally, citation searching of both reviews was carried out to potentially identify any further models published since 2008.

Development of search strategy

The purpose of the review was not to identify every single model for cardiovascular disease but rather to identify potential model structures that could be adapted or used to help construct the RUPEE NHS model. Initially, it was planned that an updated search using the search strategy devised by Unal et al. (2006) and updated by Capewell et al. (2008) would be used. However, the purpose of both reviews had been to identify notable policy models at the population level. It was felt that redoing this review could potentially fail to return other models which could be used such as those developed for NICE guidelines. The choice of databases was discussed with a systematic reviewer based at HERG. The search strategy was carried out using the following databases:

- NHS economic evaluation Database (NHS EED): this database contains economic evaluations of healthcare interventions and is produced by the NIHR Centre for Reviews and Dissemination (CRD) at the University of York, UK.
- National Institute for Health Research (NIHR) Health Technology Assessment (HTA) monograph series: This series publishes research about the effectiveness, costs and broader impact of healthcare treatments and tests (within a UK National Health Service (NHS) setting).
- National Institute for health and care excellence (NICE) website: this database publishes evidence based guidance on preventative, diagnostic and treatment interventions for disease and ill health.

NHS EED was identified as an appropriate database as this database reviews and produces critical commentaries economic evaluations of 'key' relevance to the UK NHS. The critical commentaries provide a summary of the overall reliability and generalisability of the study. The NICE HTA monograph series publishes research including cost-effectiveness analyses of healthcare treatment and tests; the series was searched to identify published HTA's which have developed or used a cardiovascular disease model. The NICE guidelines website was searched to identify guidelines related to cardiovascular disease (for example guidelines for lipid modification).

The search terms used in the search included 'cardiovascular disease', 'coronary heart disease', 'stroke', 'myocardial infarction', 'angina' and 'peripheral artery disease'. Appendix 1 contains further details of the searches carried out in each database.

Exclusion criteria

Studies were excluded if they did not discuss the development or review of an economic model; if no disease states for cardiovascular disease were included in the model; if the focus of the study was a diagnostic test or surgical intervention where the focus of the evaluation was a short term follow up, (<10 years). Studies were not excluded on the basis of intervention (treatment or lifestyle intervention) or on the basis of date published or language.

Data extraction form

The use of a standard checklist such as the Drummond economic evaluation checklist was considered to review each study but was found to be unsuitable for reviewing the models as the design of the checklist leads the reviewer to evaluate the cost-effectiveness analysis inputs and outcomes with only two questions referring to the model structure: regarding the choice and details of the model. (3)

Therefore, a data extraction form was designed to extract data that was required to meet the purpose of the review. An initial data extraction form was developed which extracted data on the following items:

- Paper (Author, Year)
- Purpose of the Model
- Setting and Population
- Interventions
- Type of model (Simulation, Markov Model, other)
- Brief description of Model
- Cardiovascular disease risk algorithms

- Risk factors included to calculate cardiovascular disease risk
- Disease stages (Health states) included in model
- Source of data inputs used in model (Population data, mortality rate, treatment uptake and effectiveness, other)
- Probabilistic Distributions and Parameters

The form was refined further to only extract data which was relevant for this review. As the purpose of the review was to inform the model structure and design the extraction of data inputs and probabilistic distributions and parameters were removed from the data extraction form. The initial data extraction form also extracted data on the quality of each model. An assessment of quality criteria for models has been suggested in guidelines from the International Society for Pharmaeconomics and Outcomes Research (ISPOR). (4,5) The systematic review by Unal et al. used the guidelines suggested by ISPOR to create a grading system for model papers based on the sensitivity, validity and transparency of a model. As the purpose of the review is not to evaluate inputs, the form was further refined and information on sensitivity analyses were not extracted. However, the data extraction forms did extract information on whether the model had been validated (including details of validation). The refined data extraction form also included a section on whether the model had been adapted for further studies. An example of a completed data extraction form can be found in Section 2.

Categorisation of Models

Each model was categorised (modelling approach) based on the taxonomy of model structures as developed by Brennan et al. (6)

Findings

The majority of models identified for review used a state transition approach (13 models) with five models adopting a hybrid state transition, in all cases a hybrid Markov-simulation model. (7-24) Only one decision tree model was identified, whereas 10 individual simulation model were identified. (25-35) Another popular approach was to use a systems dynamics modelling approach (5 models).(36-40) Other modelling approaches identified included an age period cohort (APC) approach (1 model); a tabular cell based model used by the World Health Organisation to estimate the global burden of disease; two life table approaches; a mathematical stroke epidemiological model and the Archimedes model which uses a method based on Fourier expansions using standard mathematical techniques to simulate individuals (proprietary model). (41-46)

The identified models categorised according to modelling approach can be found in Table 1.

The purpose of the review was to identify the best modelling approach for the RUPEE study. To this end, we reviewed the models to identify advantages and disadvantages of each approach. Details of the advantages and disadvantages of the two main modelling approaches used (Markov models and Simulation models) can be found in the paper associated with this supplementary appendix. Table 2 presents a summary of six models which used a different modelling approach (decision tree, state transition model, simulation model, systems dynamic and hybrid model).

Additionally, schematic illustrations of several models were used to aid discussions about the different types of modelling approaches with clinical experts. Figure 1 in Section 2 is an example of the schematic illustration for the model developed for the NICE clinical guidelines 181 which evaluated statin treatment in primary and secondary care. (47)

Table 1- Models identified in Literature Review Search

Life Table/Cell base/Tabular model	Decision Tree	State Transition	Hybrid model	Simulation	Systems Dynamic/ Compartmental model
WHO Global Burden of Disease (42)	Whitfield et al. (UK) (25)	Grover et al. CVD Life Expectancy model (Canada)- Markov Model (7)	Rotterdam Ischemic disease and stroke (RISC) model<br>Markov model structure with individual simulation (20)	Southampton CHD Policy Analysis Model 'Treatment' – individual simulation (26)	Weinstein et al. CHD heart disease policy model (USA) (36)
Schau et al. Stroke Model (Denmark)(45)		Stroke Treatment Economic Model (STEM)- USA (8)	Duke Stroke Policy and Prevention Model USA (SPPM)<br>Semi-Markov/simulation model (21)	CHD Policy Analysis Model 'Prevention'- individual simulation (27)	IMPACT model (including adaptations of model) (37)
Tobias et al. APC Model (41)		RIVM Chronic Disease- Markov Model (9)	A Dynamic modelling tool for generic health impact assessments (Dynamo-HIA)<br>Markov/partial simulation model (22)	Prevent – Macro simulation model using aggregated data (policy tool) (28)	Sundberg et al.- Compartmental model (38)
Struijs et al. Dynamic multi-state life table (43)		Ward et al . (ScHAAR statins model) and adaptations- Markov Model (10) (47)	Korean Individual Microsimulation Model for Cardiovascular Health Interventions<br>Hybrid Markov/ individual simulation model (23)	Foresight Obesity Model UK – stochastic cohort simulation approach (29)	Model of Resource Utilization, Costs and Outcomes for Stroke, (MORUCOS, Australia)- Compartmental model (39)
Archimedes (USA) (46)		Smith-Spangler et al- Markov Model (11)	Soresen et al. Simulation model<br>Markov model/individual simulation (24)	POHEM- Canada, Microsimulation (30)	PopMod: a longitudinal population model with two interacting disease

					states- Compartmental model (40)
Malik et al. Life Table (44)		Newman et al. Combination polypharmacy, Markov model 12)		EUROASPIRE III health economics project- Individual simulation (31)	
		Grosso et al, Markov Model 13)		OECD and WHO microsimulation chronic disease prevention simulation model- microsimulation (32)	
		Gillespie et al. SPHERE Markov Model (14)		Ara et al. Obesity model- Cohort simulation (33)	
		Wisloff et al. NorCaD Markov Model (15)		Department of Health Vascular Checks Model- Simulation (34)	
		Nash et al. Markov Model (16)		Green et al. Chronic Disease Policy Model- Discrete Event Simulation (35)	
		Lovibond et al. Markov Model (17)			
		Greving et al. Markov Model (18)			
		NICE Clinical Guidelines CG127 (19)			

WHO- World Health Organisation, APC- Age Period Cohort, CHD- Coronary heart disease, OECD- Organisation for Economic Co-operation and Development, ScHAAR- School of Health and Related Research, NICE- National Institute for Health and Care Excellence

Table 2- Summary of CVD models

Model name (Author)	Model Type	Risk factors	Health States/Events	Transparency & Validation	Limitations
Stroke Model (Whitfield et al.) (25)	Decision Tree	BMI, Type II diabetes, smoking, total and HDL cholesterol, SBP	Acute episode included: Acute CVD, Elective CVD, Heart Failure, Renal replacement procedures, Stroke, Diabetes (hypoglycaemia)	Internal validation: predicted number of CVD related admissions based on risk factor data compared to actual data (from five UK primary care trusts)- found results to be accurate<br><br>No external validation conducted	The model uses an aggregate approach despite having individual data<br><br>Short time frame also used, suitable for decision tree but potentially if a longer time frame was used this would not be a suitable model
NICE lipid modification guidelines economic model (CG181) (47)	Markov Model	Not explicitly stated	Death from cardiovascular cause and non CVD death, stable angina, unstable angina, myocardial infarction, transient ischaemic attack, heart failure, peripheral artery disease and post event states for each non-fatal event	Yes, the model structure, assumptions and inputs are clearly reported<br><br>Validation has not been stated, this is an update of a previously widely used model (ScHAAR statins model NICE TA94) (10)	The model is limited by the Markovian assumption of memoryless though it does have tunnel (post event) states. The cohort can experience each event only once<br><br>The model structure is not suitable to simulate a heterogeneous population
RISC state transition hybrid model (20)	Hybrid Markov Model	sex, age, smoking status, SBP & DBP, BMI, waist to hip ratio, ankle-brachial index, levels of plasma glucose, total cholesterol,	Well, Stroke, CHD, CHD & Stroke, Other Death, CVD death	Internal validation: cumulative incidences simulated by RISC model compared to Rotterdam study incidences- similar.	Allow for individual heterogeneity to be modelled, but limited by Markovian state transition model (progression

		HLD, creatinine, family history CVD, hypertension, taking antihypertensives or BP over 160/90, presence diabetes II, intermittent claudication, angina, AF, TIA or prevalent CVD		External validity tested- used NORFOLK EPIC dataset and simulation incidences using model- incidences similar	between states and handling of time). Could potentially be slow computationally to run (uses six transition probabilities equations per individual) if more health states or risk factors are required
IMPACT (Capewell et al) (37)	Compartmental/ systems dynamics model	Cigarette smoking, total cholesterol, systolic blood pressure, BMI, diabetes, physical activity and fruit and vegetable consumption	Deaths prevented or postponed from reductions in risk were the main CHD outcome<br><br><u>Nine patient groups were evaluated:</u><br><br>Patients treated in hospital for acute myocardial infarction (MI)<br><br>Patients admitted to hospital with unstable angina<br><br>Community dwelling patients who have survived a MI >1yr<br><br>Patients who had undergone a previous	A technical appendix was provided a recent paper which used the IMPACT model and this provided detailed information on the equations used to estimate deaths prevented or postponed from a treatment intervention or a reduction in CVD risk factors and provided all data sources that were used in the modelling	Cost and QALYs were not Considered<br><br>The model did not look at th reduction in CVD events, it w limited to avoided mortality from CHD<br><br>A recent expansion of the m (IMPACT 2) is available, how though online this model is a black box and a technical appendix was not available<br><br>IMPACT2 is a DES model

			revascularisation procedure<br><br>Community dwelling patients with coronary artery disease<br><br>Patients admitted to hospital with heart failure<br><br>Community dwelling patients with heart failure<br><br>Hypercholesterolaemic patients without CHD<br><br>Hypertensive patients without CHD		
CHD Policy Analysis Model – Prevention component (Babad et al). (27)	Discrete Event Simulation	Age, sex, SBP, total cholesterol and smoking	Onset of stable angina, unstable angina, myocardial infarction, sudden cardiac death, stroke death, other cardiovascular disease, cancer death and death from other or unknown cause (potential to include HDl cholesterol)	The model structure could be replicated – however no data inputs are given regarding treatment effectiveness	Use of Framingham study to estimate baseline risk- recent studies have shown that QRISK is more suited to a UK population<br><br>Computational requirements: Model was run in special software (POST, DELPHI framework). This type of model would be computationally intensive to run in widely available

					packages such as Microsoft Excel
Department of Health Vascular checks economic model (34)	Individual simulation	Age, gender, townsend score, BMI, SBP, Smoking status, Total cholesterol/HDL ratio, record family history of CHD	Not explicitly stated. Costs and health benefits applied in the model were based on published NICE guidance (PH1002, CG43, CG34 and TA94)	The model inputs and data inputs are clear	Cost and QALYs relating to interventions were not directly estimated: rather they were sourced from existing guidance and linked to the simulation outputs<br><br>Requirement for a suitable large dataset to simulate can be expensive. The Department of Health used the proprietary GP database QRESEARCH (approximate cost of dataset £15-20,000).

BMI-body mass index, HDL- high-density lipoprotein, SBP- systolic blood pressure, DBP- diastolic blood pressure, CVD-cardiovascular disease, CHD-coronary heart disease, BP- blood pressure, AF- atrial fibrillation, TIA- transient ischemic attack, NICE-National Institute for Health and Care Excellence, DES-discrete event simulation, MI-myocardial infarction, ScHAAR-School of Health and Related Research, RISC- Rotterdam Ischemic disease and stroke model

Section 2- Example of completed data extraction form

Model name: Southampton Disease Model (CHD Policy Analysis Model) 'Treatment Model'

Paper (Author, Year): The development of a simulation model of the treatment of coronary heart disease (Keith Cooper and Ruth Davies, 2002)

Journal: Health Care Management Science 5, 259-267

<u>**Model Details**</u>

Model Structure: Discrete Event Simulation

Model software: Patient orientated simulation technique (POST) software with a Delphi interface.

Study Population & Setting: Individuals with stable angina, unstable angina or myocardial infarction (till age 85 or death).

Purpose of Model: The model is used to evaluated revascularisation at a hospital level rather than population based. Looks at progress of patients after a coronary event.

Patient characteristics: Given attributes of age, gender, vessel disease, time before cardiac death and time to age 85.

Model Description: New patients enter the model with SA, UA or MI (proportion randomly determined using incidence rate of disease). The following assumptions are employed:

- Risk of non-cardiac death
- Risk SA or UA leads to risk of MI
- SA leads to risk of UA
- Sampled time to event (MI, death, UA) depend on age and vessel disease
- Risks of UA, MI, & death increase with age, severe vessel disease and with a history of previous myocardial infarctions.
- Risks are independent of each other and are multiplied by baseline risks to change the projections of MI and death.
- Time updated Gompertz distribution (hazard function) used to estimate time to event (includes relative risks from vessel disease, prior history and interventions)

Progression to health events: Stable angina- Start in GP state receiving treatment (medical), assuming some have symptoms controlled and some not. Some are transferred to outpatient's investigations (now or in x years). After outpatients, some join a waiting list for an angiogram (queue) and those who do not go to a medical treatment stage. Vessel disease extent will determine next step after angiogram (can change this rule/input in simulation). Patients can bypass graft, angioplasty. Incidence data from Health Survey for England and GP Morbidity data.

Validation of model: validated cardiac deaths against mortality data from Office for National Statistics, based on death certificates. Model did underestimate deaths in females. Authors surmised this was due to poor reporting of causes of death on certificates.

Limitations Study (2002) does not mention the application of costs or QALYs and it looks at CHD events only. The authors noted that the model will be developed further to link the outputs to costs and to include secondary prevention such as aspirin or anti-cholesterol agents and to link the treatment with the prevention model (Prevent model developed by Babad et al.)

Summary- Discrete event simulation model for progress of patients after a coronary event. Individuals have angina and can progress to unstable angina or myocardial infarction. Changes in risks in one part can affect other parts of model. This model did allow for resource constraints such as availability of tests

References

(1) Unal B, Capewell S, Critchley JA. Coronary heart disease policy models: a systematic review. BMC Public Health 2006;6(1):213.

(2) Capewell S, Allender S, Critchley J, Lloyd-Williams F, O'Flaherty M, Rayner M, et al. Modelling the UK burden of cardiovascular disease to 2020: a research report for the Cardio & Vascular Coalition and the British Heart Foundation. British Heart Foundation 2008(Available at http://www.healthimpact.org.uk/content/resources/z154_modelling_the_burden_of_cv_disease.pdf).

(3) Drummond MF, O'Brien B, Stoddart GL, Torrance GW. Methods for the economic evaluation of health care programmes.

(4) Weinstein MC, O'Brien B, Hornberger J, Jackson J, Johannesson M, McCabe C, et al. Principles of good practice for decision analytic modeling in health-care evaluation: report of the ISPOR Task Force on Good Research Practices—Modeling Studies. Value in health 2003;6(1):9-17.

(5) Eddy DM, Hollingworth W, Caro JJ, Tsevat J, McDonald KM, Wong JB, et al. Model transparency and validation: a report of the ISPOR-SMDM Modeling Good Research Practices Task Force-7. Med Decis Making 2012 Sep-Oct;32(5):733-743.

(6) Brennan A, Chick SE, Davies R. A taxonomy of model structures for economic evaluation of health technologies. Health Econ 2006;15(12):1295-1310.

(7) Grover SA, Paquet S, Levinton C, Coupal L, Zowall H. Estimating the benefits of modifying risk factors of cardiovascular disease: a comparison of primary vs secondary prevention. Arch Intern Med 1998;158(6):655-662.

(8) Caro JJ, Huybrechts KF. Stroke treatment economic model (STEM): predicting long-term costs from functional status. Stroke 1999 Dec;30(12):2574-2579.

(9) Van Baal P, Feenstra T, Hoogeveen R, De Wit G. Cost effectiveness analysis with the RIVM Chronic Disease Model. 2005.

(10) Ward S, Jones ML, Pandor A, Holmes M, Ara R, Ryan A, et al. A systematic review and economic evaluation of statins for the prevention of coronary events. 2007.

(11) Smith-Spangler CM, Juusola JL, Enns EA, Owens DK, Garber AM. Population Strategies to Decrease Sodium Intake and the Burden of Cardiovascular DiseaseA Cost-Effectiveness Analysis. Ann Intern Med 2010;152(8):481-487.

(12) Newman J, Grobman WA, Greenland P. Combination Polypharmacy for Cardiovascular Disease Prevention in Men: A Decision Analysis and Cost-Effectiveness Model. Preventive cardiology 2008;11(1):36-41.

(13) Grosso AM, Bodalia PN, MacAllister RJ, Hingorani AD, Moon JC, Scott MA. Comparative clinical- and cost-effectiveness of candesartan and losartan in the management of hypertension and heart failure: a systematic review, meta-and cost-utility analysis. Int J Clin Pract 2011;65(3):253-263.

(14) Gillespie P, O'Shea E, Murphy AW, Byrne MC, Byrne M, Smith SM, et al. The cost-effectiveness of the SPHERE intervention for the secondary prevention of coronary heart disease. Int J Technol Assess Health Care 2010;26(03):263-271.

(15) Wisloff T, Selmer RM, Halvorsen S, Fretheim A, Norheim OF, Kristiansen IS. Choice of generic antihypertensive drugs for the primary prevention of cardiovascular disease--a cost-effectiveness analysis. BMC Cardiovasc Disord 2012 Apr 4;12:26-2261-12-26.

(16) Nash A, Barry M, Walshe V. Cost effectiveness of statin therapy for the primary prevention of coronary heart disease in Ireland. Ir Med J 2006;99(5):144-145.

(17) Lovibond K, Jowett S, Barton P, Caulfield M, Heneghan C, Hobbs FR, et al. Cost-effectiveness of options for the diagnosis of high blood pressure in primary care: a modelling study. The Lancet 2011;378(9798):1219-1230.

(18) Greving JP, Buskens E, Koffijberg H, Algra A. Cost-effectiveness of aspirin treatment in the primary prevention of cardiovascular disease events in subgroups based on age, gender, and varying cardiovascular risk. Circulation 2008 Jun 3;117(22):2875-2883.

(19) National Institute for Health and Clinical Excellence,. Hypertension: clinical management of primary hypertension in adults. 2011;Clinical Guideline 127.

(20) van Kempen BJ, Ferket BS, Hofman A, Steyerberg EW, Colkesen EB, Boekholdt SM, et al. Validation of a model to investigate the effects of modifying cardiovascular disease (CVD) risk factors on the burden of CVD: the rotterdam ischemic heart disease and stroke computer simulation (RISC) model. BMC Med 2012 Dec 6;10:158-7015-10-158.

(21) Matchar DB, Samsa GP, Matthews JR, Ancukiewicz M, Parmigiani G, Hasselblad V, et al. The Stroke Prevention Policy Model: linking evidence and clinical decisions. Ann Intern Med 1997;127(8_Part_2):704-711.

(22) Lhachimi SK, Nusselder WJ, Smit HA, van Baal P, Baili P, Bennett K, et al. DYNAMO-HIA--a Dynamic Modeling tool for generic Health Impact Assessments. PLoS One 2012;7(5):e33317.

(23) Kang H, Ko S, Liew D. Results of a Markov model analysis to assess the cost-effectiveness of statin therapy for the primary prevention of cardiovascular disease in Korea: the Korean Individual-Microsimulation Model for Cardiovascular Health Interventions. Clin Ther 2009;31(12):2919-2930.

(24) Sorensen SV, Frick KD, Wade A, Simko R, Burge R. Model-based simulation to explore the cost-effectiveness of following practice guidelines for triglyceride and low-density lipoprotein cholesterol control among patients with diabetes mellitus and mixed dyslipidemia. Clin Ther 2009;31(4):862-879.

(25) Whitfield MD, Gillett M, Holmes M, Ogden E. Predicting the impact of population level risk reduction in cardio-vascular disease and stroke on acute hospital admission rates over a 5 year period—a pilot study. Public Health 2006;120(12):1140-1148.

(26) Cooper K, Davies R, Raftery J, Roderick P. Use of a coronary heart disease simulation model to evaluate the costs and effectiveness of drugs for the prevention of heart disease. J Oper Res Soc 2008;59(9):1173-1181.

(27) Babad H, Sanderson C, Naidoo B, White I, Wang D. The development of a simulation model of primary prevention strategies for coronary heart disease. Health Care Manag Sci 2002;5(4):269-274.

(28) Gunning-Schepers L, Barendregt J, van der Maas P. PREVENT, a Model to Estimate the Health Benefits of Prevention. Models of Non-communicable Diseases.Health status and Health Service Requirements 1992:55-69.

(29) Keaver L, Webber L, Dee A, Shiely F, Marsh T, Balanda K, et al. Application of the UK foresight obesity model in Ireland: the health and economic consequences of projected obesity trends in Ireland. 2013.

(30) Manuel DG, Tuna M, Hennessy D, Bennett C, Okhmatovskaia A, Fines P, et al. Projections of preventable risks for cardiovascular disease in Canada to 2021: a microsimulation modelling approach. CMAJ Open 2014 May 20;2(2):E94-E101.

(31) Annemans L, Lamotte M, Clarys P, Van den Abeele E. Health economic evaluation of controlled and maintained physical exercise in the prevention of cardiovascular and other prosperity diseases. Eur J Cardiovasc Prev Rehabil 2007 Dec;14(6):815-824.

(32) Cecchini M, Sassi F, Lauer JA, Lee YY, Guajardo-Barron V, Chisholm D. Tackling of unhealthy diets, physical inactivity, and obesity: health effects and cost-effectiveness. The Lancet 2010;376(9754):1775-1784.

(33) Ara R, Blake L, Gray L, Hernandez M, Crowther M, Dunkley A, et al. What is the clinical effectiveness and cost-effectiveness of using drugs in treating obese patients in primary care? A systematic review. 2012.

(34) Department of Health. Economic Modelling for Vascular Checks. 2008; Available at: www.healthcheck.nhs.uk/document.php?o=225. Accessed July, 2015.

(35) Green N, Smith D, Sperrin M, Buchan I. A Novel Chronic Disease Policy Model. arXiv preprint arXiv:1009.0405 2010.

(36) Weinstein MC, Coxson PG, Williams LW, Pass TM, Stason WB, Goldman L. Forecasting coronary heart disease incidence, mortality, and cost: the Coronary Heart Disease Policy Model. Am J Public Health 1987 Nov;77(11):1417-1426.

(37) Fidan D, Unal B, Critchley J, Capewell S. Economic analysis of treatments reducing coronary heart disease mortality in England and Wales, 2000-2010. QJM 2007 May;100(5):277-289.

(38) Sundberg G, Bagust A, Terént A. A model for costs of stroke services. Health Policy 2003;63(1):81-94.

(39) Mihalopoulos C, Cadilhac DA, Moodie ML, Dewey HM, Thrift AG, Donnan GA, et al. Development and application of Model of Resource Utilization, Costs, and Outcomes for Stroke (MORUCOS): An Australian economic model for stroke. Int J Technol Assess Health Care 2005;21(04):499-505.

(40) Lauer JA, Rohrich K, Wirth H, Charette C, Gribble S, Murray CJ. PopMod: a longitudinal population model with two interacting disease states. Cost Eff Resour Alloc 2003 Feb 26;1(1):6.

(41) Tobias M, Sexton K, Mann S, Sharpe N. How low can it go? Projecting ischaemic heart disease mortality in New Zealand to 2015. N Z Med J 2006;119(1232).

(42) Murray CJ, Lopez AD. Alternative projections of mortality and disability by cause 1990–2020: Global Burden of Disease Study. The Lancet 1997;349(9064):1498-1504.

(43) Struijs JN, van Genugten ML, Evers SM, Ament AJ, Baan CA, van den Bos, Geertrudis AM. Modeling the future burden of stroke in the Netherlands impact of aging, smoking, and hypertension. Stroke 2005;36(8):1648-1655.

(44) Malik IS, Bhatia VK, Kooner JS. Cost effectiveness of ramipril treatment for cardiovascular risk reduction. Heart 2001 May;85(5):539-543.

(45) Schau B, Boysen G, Truelsen T, Boden-Albala B, Cheng J, Babamoto E, et al. Development and validation of a model to estimate stroke incidence in a population. Journal of Stroke and Cerebrovascular Diseases 2003;12(1):22-28.

(46) Schlessinger L, Eddy DM. Archimedes: a new model for simulating health care systems—the mathematical formulation. J Biomed Inform 2002;35(1):37-50.

(47) National Institute for Health and Care Excellence (NICE). Lipid modification: cardiovascular risk assessment and the modification of blood lipids for the primary and secondary prevention of cardiovascular disease. 2014; Clinical Guideline 181.
