# Supplementary material for: A protocol for an economic evaluation of a polypill in patients with established or at high risk of cardiovascular disease in a UK NHS setting: RUPEE (NHS) study
Source: BMJ Open. 2018 Mar 14;8(3):e013063. doi: 10.1136/bmjopen-2016-013063 (PMC5857692; doi:10.1136/bmjopen-2016-013063)
Supplement: Author's manuscript [file bmjopen-2016-013063.draft_revisions.pdf]

A Protocol for a modelled economic evaluation to evaluate a polypill in patients with established or at high risk of cardiovascular disease: Researching the UMPIRE Processes for Economic Evaluation in the UK National Health Service - RUPEE (NHS)

Journal:	*BMJ Open*
Manuscript ID	bmjopen-2016-013063
Article Type:	Protocol
Date Submitted by the Author:	16-Jun-2016
Complete List of Authors:	Crossan, Catriona; Health Economics Research Group<br>Moulay Dehbi, Hakim; Imperial College London, Department of Epidemiology and Biostatistics<br>Thom, Simon; Imperial College London, International Centre for Circulatory Health<br>Poulter, Neil; Imperial College London<br>Williams, Hilarie; Imperial College London, International Centre for Circulatory Health<br>Rodgers, Anthony; The George Institute for Global health,<br>Jan, Stephen; The George Institute for Global Health,<br>Lord, Joanne; University of Southampton, Health Technology Assessments Centre
**Primary Subject Heading**:	Health economics
Secondary Subject Heading:	Cardiovascular medicine, Patient-centred medicine
Keywords:	Coronary heart disease < CARDIOLOGY, HEALTH ECONOMICS, Coronary intervention < CARDIOLOGY

SCHOLARONE™
Manuscripts

A Protocol for a modelled economic evaluation to evaluate a polypill in patients with established or at high risk of cardiovascular disease: *Researching the UMPIRE Processes for Economic Evaluation in the UK National Health Service* - RUPEE (NHS)

Authors: Crossan C, Dehbi HM, Thom S, Poulter N, Williams H, Rodgers A, Jan S, Lord J

Corresponding author: Catriona Crossan, Health Economics Research Group, Brunel University London, Uxbridge, Middlesex, UB8 3PH, United Kingdom. catriona.crossan@brunel.ac.uk Tel: 0044 (0) 1895 267920. Fax:0044 (0) 1895 269708

Author Affiliations

Hakim-Moulay Dehbi. International Centre for Circulatory Health, Imperial College, London, W2 1LA, United Kingdom.

Simon Thom. Peart-Rose Research Unit, International Centre for Circulatory Health NHLI, Imperial College London (Hammersmith Campus), Du Cane Road, London, W12 0HS, United Kingdom.

Neil Poulter. Peart-Rose Research Unit, International Centre for Circulatory Health NHLI, Imperial College London (Hammersmith Campus), Du Cane Road, London, W12 0HS, United Kingdom.

Hilarie Williams. Peart-Rose Research Unit, International Centre for Circulatory Health NHLI, Imperial College London (Hammersmith Campus), Du Cane Road, London, W12 0HS, United Kingdom.

Anthony Rodgers. The George Institute for Global Health, University of Sydney, Camperdown, New South Wales, Australia

Stephen Jan. The George Institute for Global Health, University of Sydney, Camperdown, New South Wales, Australia

Joanne Lord. Southampton Health Technology Assessments Centre, University of Southampton, 1st Floor, Epsilon House, Enterprise Road, Southampton Science Park, Southampton SO16 7NS

Keywords: Cardiovascular disease, polypill, adherence, cost-effectiveness, economic evaluation

Word Count 4,784

Figures 2 **Tables** 1

For peer review only - http://bmjopen.bmj.com/site/about/guidelines.xhtml

Abstract

Introduction: The 'Use of a Multi-drug Pill in Reducing cardiovascular Events' (UMPIRE) trial was a randomised controlled clinical trial evaluating the impact of a polypill strategy on adherence to indicated medication in a population with established cardiovascular disease of or at high risk thereof. The aim of RUPEE-NHS is to estimate the potential health economic impact of a polypill strategy for CVD prevention within the NHS using UMPIRE trial and other relevant data. This paper describes the design of a modelled economic evaluation of the impact of increased adherence to the polypill versus usual care amongst the UK UMPIRE participants.

Methods and Analysis: As recommended by ISPOR modelling guidelines a review of published CVD models was undertaken to identify the most appropriate modelling approach and structure. The review was carried out in the electronic databases, MEDLINE and EMBASE. 40 CVD models were identified from 22 studies, the majority of economic models were health state transition cohort models and individual level simulation models. The findings were discussed with clinical experts to confirm the approach and structure. An individual simulation approach was identified as the most suitable method to capture the heterogeneity in population CVD risk. RUPEE-NHS will use UMPIRE trial data on adherence to the polypill versus usual care to estimate the long term cost-effectiveness of the polypill strategy.

Dissemination: The evaluation findings will be presented in open access scientific and healthcare policy journals and at national and international conferences. We will also present findings to NHS policy makers and pharmaceutical companies.

Strengths and Limitations

This paper provides a clear outline of how a model for an economic evaluation is developed.

Providing an outline of the model structure which includes details on the underlying epidemiology and data inputs will add transparency to the findings of the RUPEE-NHS study

Though the model has been designed to include all major adverse and beneficial effects of treatment, the model structure will not include every potential treatment effect, for example the benefits of treatment on Alzheimer's disease will not be included.

For peer review only - http://bmjopen.bmj.com/site/about/guidelines.xhtml

INTRODUCTION

Adherence to recommended preventive medication regimes (1,2) in people at high risk of cardiovascular disease (CVD) is low, even in high income countries. (3) Poor adherence is associated with greater deterioration in health status and increased health care costs (4) and studies have shown that improved adherence to medication is associated with clinical benefits.(5) CVD preventive medication typically involves several drugs and adherence is inversely proportional to the number of prescriptions. Furthermore, physician inertia and patient resistance present barriers to initiating or restarting full recommended therapy. A single pill that includes several indicated drugs (a "polypill"), may improve long-term adherence by addressing these issues. If the polypill is priced lower than the price of the pills bought separately, it will also make it more affordable. (6,7) The UMPIRE (Use of a Multidrug Pill in Reducing Cardiovascular events) clinical trial was set up to evaluate the polypill in patients with or at high risk of CVD.

The UMPIRE trial randomised 2004 participants with established CVD (prior CVD event such as stroke or myocardial infarction) or at high risk of CVD (defined as a 5 year risk of >15%) based in India, England, Ireland and The Netherlands to either the polypill or usual care. The primary outcome of the trial was adherence to indicated treatments (statin, aspirin and two blood pressure lowering drugs). Other outcomes included systolic blood pressure (SBP) and low-density lipoprotein cholesterol (LDL-C). The trial found that the use of a polypill strategy resulted in greater adherence to treatment at 15 months and significant improvements in SBP and LDL-C. Detailed results and a description of the UMPIRE trial protocol are available. (8, 9)

UMPIRE collected data on resource use and self-reported health related quality of life using the EQ-5D. In order to estimate the long term costs and health outcomes associated with the polypill strategy an economic model is required. Due to differences in the patient population, care pathway and health care costs, separate analyses are needed for the four participating countries.

The analysis of the UMPIRE English trial data, (Researching the UMPIRE Processes for Economic Evaluation in the National Health Service (RUPEE-NHS)), aims to estimate the cost-effectiveness of the polypill strategy compared to conventional multi-drug therapy for the prevention of established cardiovascular disease in English NHS patients with or at high risk of CVD. The RUPEE (NHS) study will use UMPIRE English trial data on adherence to the polypill and will develop an economic model to estimate cost effectiveness.

The aim of this paper is to detail the modelling plan for the RUPEE (NHS) study.

For peer review only - http://bmjopen.bmj.com/site/about/guidelines.xhtml

METHODS

Model design process

An economic model has been described as a mathematical framework that represents reality at an adequate level of detail to inform clinical or policy decisions. (10) Guidelines on modelling produced by the International Society for Pharmacoeconomics and Outcomes Research (ISPOR) recommend that it is best practice to carry out a conceptualisation process prior to programming the economic model. This process has two distinct components: specification of the study question and economic model. (11)

Specification of the study question

The first component informs choices about how to structure the economic model and parameters. The RUPEE (NHS) study aims to evaluate two different treatment strategies in a population with or at high risk of CVD. The population for the economic model is defined by the inclusion criteria of the UMPIRE trial. (9) The inclusion criteria are listed below:

- Aged ≥18 years and
- High CVD risk defined as either established atherothrombotic CVD (history of coronary heart disease (CHD), ischaemic cerebrovascular disease, or peripheral arterial disease (PAD)) or a 5 year risk of ≥15% calculated using the Framingham risk equation

The economic model will evaluate the polypill strategy compared to usual medication. In the UMPIRE trial, participants assigned to the polypill received one of 2 versions: version 1 contained aspirin 75mg, simvastatin 40mg, lisinopril 10mg and atenolol 50mg, and version 2 contained the same ingredients but substituted hydrochlorothiazide 12.5mg for atenolol 50mg. Participants assigned to usual care continued taking medications as prescribed by their general practitioner (GP).

The RUPEE (NHS) study will follow guidelines for modelling health technologies as recommended by the National Institute for Health and Care Excellence. (NICE) (11) Therefore a NHS and Personal Social Services (PSS) perspective will be adopted to measure health service resource use and health related quality of life will be measured by quality adjusted life years (QALYs) obtained using the EQ-5D. As per the NICE guidelines, costs and QALYS will be discounted at a rate of 3.5% per year. (12) The time horizon reflected in the economic model will be lifetime to represent the chronic nature of CVD.

For peer review only - http://bmjopen.bmj.com/site/about/guidelines.xhtml

Conceptualisation of the economic model

The second component of the conceptualisation process involves defining the economic model. There are two steps to this approach. The first step is to identify the appropriate modelling approach. The modelling approach defines the analytical framework of the economic model. Different types of analytical frameworks have been used to represent CVD including decision trees, state transition models, compartmental models, individual simulation models and hybrid models which often combine elements from different frameworks. (13-17)

The second step determines the underlying structure of the analytical framework, which will represent the disease and care pathway. The modelling approach needs to reflect: 1) CVD disease and care pathway for this population; 2) the beneficial and adverse effect of treatment (polypill or usual care); 3) the impact of increased adherence to treatment on health outcomes.

Guidelines produced by ISPOR on modelling recommend that existing models addressing related problems should be reviewed as this approach can help identify both the modelling approach and underlying structure.(11) To inform the RUPEE (NHS) economic model, we carried out a review of published models evaluating interventions for CVD.

Review of published CVD economic models

The purpose of the literature review was to identify the appropriate analytical framework to represent the decision problem. The literature review also aimed to inform the underlying model structure: disease and care pathway.

Search strategy

The search strategy was conducted using the NHS Economic Evaluation Database (NHS EED), the NIHR Health Technology Assessment (HTA) monograph series and the NICE guidelines website. The search terms used included ‘cardiovascular disease’, ‘coronary heart disease’, ‘stroke’, ‘myocardial infarction’, ‘angina’ and ‘peripheral artery disease’. Studies were excluded from the review if they did not discuss the development or review of an economic model; if no disease states for cardiovascular disease were included in the model; if the focus of the study was a diagnostic test or surgical intervention where the economic model used a time frame of <10 years. Studies were not excluded on the basis of intervention (drug treatment or lifestyle intervention) or on the basis of date published or language. We developed a data extraction form which included fields on model purpose, structure, health states and events, transparency and validation. We did not collate

For peer review only - http://bmjopen.bmj.com/site/about/guidelines.xhtml

information about the findings of the model as the objective of the review was to identify alternative model frameworks and methods used to represent CVD.

An initial general literature search identified a 2006 systematic review of CHD policy models by Unal *et al*. which was updated in 2008 by Capewell *et al*. and expanded to include stroke models. (17,18) The review by Capewell *et al*. identified seven 'notable' CHD models (of which six had been identified in the previous review by Unal *et al.*), nine stroke models and several models that were currently in development at the time of publication. We reviewed the notable models and models in development identified by Capewell *et al*. Citation searching of both systematic reviews was carried out to identify other models published since 2008.

Review findings

Overall 22 studies were identified which reported on 40 CVD models. Figure 1 presents the flowchart for the search strategy. The searches found several studies which reported on the same model, for example the IMPACT CHD model developed by Capewell *et al*. was used in analyses of CVD in other populations. (19) In some cases, a model was adapted for different analyses, such as the Sheffield model which was developed to evaluate statin therapy and was then adapted for use in the development of the NICE guidelines for lipid modification. (1,20) The Sheffield model was also partially used in a whole population modelling study by Barton *et al*. (13)

[Figure 1- Flowchart for search strategy]

Modelling approach

The search identified that the two most commonly used modelling approaches were health state transition cohort models and individual-level simulation models. Both approaches were critically assessed to determine their suitability to capture the disease and care pathway.

A cohort model can be defined as any model which estimates the outcomes for a group of patients, whereas with a patient level simulation, outcomes are evaluated at the individual level. Therefore, one of the main differences between the two approaches is how they estimate costs and QALYs: cohort models estimate expected costs and QALYs for the modelled population as a whole, whereas individual level simulation models estimate cost and QALYs for each individual and the average is taken across the sample.

With a health state transition cohort model, the population progresses through a set of mutually exclusive health states at regular intervals called cycles, determined by a predefined transition matrix. Health state transition cohort models are also commonly called Markov models. However,

For peer review only - http://bmjopen.bmj.com/site/about/guidelines.xhtml

such models are only Markovian when they display the Markovian 'memoryless' property where the progression of the patient through the model is only dependent on the current state in which the patient resides and not on anything that happened before they entered that health state. It is also possible to model at the individual level using a state transition model by sampling probabilities for each individual patient to experience a particular transition in each model cycle. (21)

Both model approaches can use a discrete time approach: with this approach the model cycle length will be defined in advance. The cohort or individual progress through health states or events which represent the disease pathway and only one event may occur within each cycle length. Costs and QALYs are updated once per cycle. Alternatively, individual level simulation models are often set up as discrete event simulations (DES). With a DES approach, an event can occur at any time point, for example, an event could occur at three months, one year and twenty years. As an event occurs, costs and QALYs are recorded and updated for each individual.

A health state transition model was used to develop NICE guidance for lipid modification treatment. (1) The limitation of this approach is that it may be unable to capture the underlying heterogeneity in the population. Individual CVD risk can be estimated using CVD risk algorithms such as QRISK2 which use a range of patient characteristics such as age, sex, ethnicity, systolic blood pressure and body mass index to estimate a 10 year CVD risk.(22) To capture this complexity in a health state transition model would require the construction of a large number of subgroups to reflect different subsets of patient characteristics and the variation in CVD risk in the population. This could become impractical to model. It also has the disadvantage that accuracy could be lost by using representative values for subgroups. An individual simulation model structure may be more appropriate to model the level of detail required to estimate CVD outcomes reflective of those in the population.

The Markovian memoryless property means that data on individual patients is not retained as patients progress through the model. It is possible to overcome this by adding tunnel states to capture changes in patient characteristics. However, this could make the model very complex and impracticable to run as a large number of these tunnel states may be required.

To accurately identify the effectiveness of each treatment strategy in a population with or at high risk of CVD, an individual simulation model was deemed the most appropriate for the RUPEE (NHS) study to reflect the heterogeneity in the population which impacts on the risk of a CVD event and

For peer review only - http://bmjopen.bmj.com/site/about/guidelines.xhtml

subsequent costs and outcomes. The individual simulation model will use a discrete event approach to handle time.(21)

Model structure

The findings of the review were discussed with clinical experts to confirm the health events and the methods used to model the progression of persons through the disease pathway.

Model events (CVD, diabetes and adverse events)

The most commonly included types of CVD events in the reviewed models were CHD (angina and myocardial infarction), cerebrovascular events (transient ischaemic attack (TIA) and stroke) and PAD. It was decided that the CVD events relevant for the current model would reflect those most commonly included in prior such models. PAD will not be included as a CVD event in the model as there is less likely to be a definable acute PAD event compared to other CVD events such as MI and stroke. We will assume that patients can experience more than one CVD event in their lifetime. The risk of CVD will also be assumed to change with age in the model.

Diabetes is a risk factor for CVD with a substantial cost and impact on health related quality of life, therefore diabetes will be included as a comorbidity in the model. The risk of new onset diabetes will be estimated using the QDiabetes risk algorithm.(23)

Adverse effects from treatment will include an increase in the risk of new onset diabetes resulting from treatment with statins and antihypertensive drugs. (24-27) The risk of a persistent cough resulting from treatment with angiotensin-converting-enzyme inhibitors (ACE inhibitors) will be included as an event. The probability of a cough resulting from treatment will be sourced from meta-analyses of randomised controlled trial (RCT) data for ACE inhibitors. As aspirin use is associated with an increased risk of gastrointestinal bleeding,(28,29) an increased risk of gastrointestinal bleeding from treatment with aspirin will be included.

Renal impairment will not be included in the model as an adverse effect of ACE inhibitors. Whilst ACE inhibitors may cause an acute rise in serum creatinine in a few patients with renal artery stenosis and more generally cause a slight short term increase in creatinine levels, the effects are complex and there may be a net improvement in renal function overall in a treated population. The rate of falls and fractures will be estimated not to alter, given the evidence from randomised trials of blood pressure lowering agents, although this is an area of debate with regard to patients with higher levels of frailty. (30, 31)

For peer review only - http://bmjopen.bmj.com/site/about/guidelines.xhtml

For peer review only

Other adverse effects from statin treatment such as liver dysfunction and myopathy will not be included in the model as these cases are rare and are assumed to have a minimal impact on outcomes. (1) (32)

Treatment with antihypertensives is associated with a reduction in heart failure, therefore this will be included as an outcome in the model. (33) Other outcomes of treatment are likely but will not be included – for example a reduction in cancer with aspirin use of more than 5 years. (34)

Progression of individuals through model

The progression of persons through the disease pathway differs depending on the modelling approach: health state transition models such as the Markov model developed for NICE guidelines on lipid modification use a predefined transition matrix to determine progression through the CVD health states.(35) Alternatively, simulation models can use risk algorithms to estimate the probability of CVD events or new onset diabetes. The NICE guidelines for lipid modification recommend the use of QRISK2, which is a risk algorithm derived to estimate primary CVD risk in UK populations. (1,22) The QRISK2 risk algorithm predicts the risk of a 10 year CHD event (angina, MI) or a cerebrovascular event (TIA, stroke). It does not include the risk of PAD. An alternative CVD risk algorithm is the Framingham equation;(36), however, a validation study comparing QRISK2 and Framingham found that QRISK2 is better calibrated to a UK population.(37) The RUPEE (NHS) model will therefore use the QRISK2 risk algorithm.

RUPEE (NHS) economic model

Figure 2 depicts the flowchart of the RUPEE (NHS) model structure. The oval shapes represent data inputs to the model, whereas the rectangular shapes represent processes.

[Figure 2 - Flowchart of RUPEE (NHS) model structure]

Model description

In the RUPEE (NHS) model costs and QALYs are recorded for each individual and an average cost and QALY for the simulated population are estimated. The RUPEE (NHS) model will be run twice, once to simulate costs and QALYs under usual care and once to simulate costs and QALYs under the polypill strategy. Individuals representing the UMPIRE trial inclusion criteria will enter the model (label 1 in Figure 2), and their baseline risk of an event will be estimated using the QRISK2 CVD risk algorithm or the QDiabetes algorithm (label 2 in Figure 2). For each individual, whether or not they are adherent to medication will be simulated using Monte Carlo simulation based on the probability of adherence in usual care (label 3a in Figure 2). If the individual is simulated to be adherent to medication their risk of a CVD event will be modified by a treatment effect (label 4 in Figure 2). For simulated

For peer review only - http://bmjopen.bmj.com/site/about/guidelines.xhtml

adherent individuals taking the polypill, the risk of an event will be further modified by the relative risk of adherence for polypill versus usual care. The relative risk of adherence for polypill versus usual care will be sourced from the UMPIRE trial data (label 3b in Figure 2). Individuals may experience a CVD event or onset of diabetes based on their estimated CVD and diabetes risk, which will be estimated using the QRISK2 and QDiabetes algorithms. Individuals may also experience an adverse reaction to medication (if adherent) including gastrointestinal bleeding, early onset of diabetes and a persistent cough. Costs and QALYs will be recorded for each event. Individuals can experience more than one event (model run for lifetime horizon) and patient characteristics such as age and history of previous events, such as a stroke or new onset diabetes, are updated during the model run, with an ensuing reflective increase in the risk of an event.

Input parameters

Each point in the flowchart is labelled and a description of the process or data requirement label is described below. Table 1 provides further details on data input parameters for the RUPEE-NHS model and potential sources of data.

1. Population Dataset

We will use the 2011 Health Survey for England (HSE) as a population dataset for the economic model. The HSE is a cross sectional survey which contains anonymised information on a representative sample of the population. The 2011 HSE dataset collected information on CVD, including individual CVD events and medication history. The dataset also contains information on demographic and socio-economic characteristics and health related data such as body mass index (BMI), SBP and LDL-C and history of CVD events. These data are required in order to estimate individual baseline risks of CVD and diabetes in the model.

2. Calculation of baseline risks of events without treatment

Baseline risks for CVD for each sampled individual will be calculated using published risk algorithms. As per recent NICE guidance for lipid modification, we will use the recommended algorithm for CVD risk, QRISK2. (1,21) The algorithm was derived using QRESEARCH, a large database derived from the pseudonymised health records of over 13 million patients registered with a general practitioner in the UK. If an individual has established CVD (previously experienced a CVD event), we will estimate a secondary CVD risk using the REACH algorithm. (38) A baseline risk for the onset of diabetes will be estimated using the QDiabetes algorithm. (23)

For peer review only - http://bmjopen.bmj.com/site/about/guidelines.xhtml

3a. Simulating adherence to treatment under usual care

The RUPEE study will evaluate the effect of adherence to medication on long term costs and health outcomes. The average rates of adherence in clinical trials can be higher than in actual practice (4) as seen in the UMPIRE clinical trial population which had an atypically high baseline adherence rate. Instead, adherence rates to medication (antihypertensives (AHT), statins and aspirin) under a usual care setting will be sourced from the 2011 HSE dataset. This will reflect adherence to medication in a usual care population. The data will be used to estimate the probability of each person being adherent or not to medication. Individual characteristics will be used as predictors of adherence: the characteristics will be chosen by referring to studies which have assessed predictors of adherence in persons taking treatment for CVD. A generalized linear mixed regression model will be used to estimate the probability of adherence to medication for each individual. The probability of persistence with medication will not be assumed to be constant, and the model will include a probability of ceasing medication over time. The probability of medication cessation will be sourced from published literature on adherence.

3b. Estimate relative risks of adherence for polypill versus usual care

We will estimate the relative risk of adherence for the polypill strategy versus usual care. A generalised linear mixed regression model will be applied to the UMPIRE trial dataset, with adherence to medication indicated as taking ≥ 2 antihypertensive drugs, a statin and aspirin for at least four days in the week prior to a recorded visit. The definition of adherence reflects that used in UMPIRE. (8)

4. Adjust risk of events for treatment

We will source data on the treatment effects of statins, antihypertensives and aspirin from meta-analyses of intention-to-treat RCTs. Intention-to-treat analyses account for non-adherence in their findings, and therefore underestimate the impact of treatment on event risk. To overcome this we will carry out sensitivity analyses to test the impact of adjusting for adherence within the trial. The risk of a CVD event will be adjusted by the relative risk of treatment, based on the treatment the person is taking and whether or not they are adherent to medication.

5. Simulation of events

Individuals in the model can experience a CVD event at a rate governed by their calculated baseline risk (estimated by the QRISK2 or REACH algorithms) and adjusted for treatment effects if they have been simulated as adherent to treatment. CVD events will be categorised as a TIA, stroke, MI or

For peer review only - http://bmjopen.bmj.com/site/about/guidelines.xhtml

angina. The relative incidence of each CVD event will be determined using published incidence data.(39) Similarly, the risk of new onset diabetes will be calculated using the QDiabetes algorithm. We will simulate the incidence of adverse events as a result of treatment: new onset diabetes and gastrointestinal bleeds. Data on the probability of an adverse event will be sourced from meta-analyses of randomised controlled trials for the relevant drugs. Mortality risk will be modelled as mortality from stroke and MI and other cause mortality. Data on other cause mortality will be estimated using national life tables for England and Wales. (40)

6. Assign cost and quality of life values

Costs and QALYs associated with each individual's simulated lifetime profile of CVD and related care will be estimated. Costs and quality adjusted life years (QALYs) will accrue for each person to reflect events, such as a stroke or new onset diabetes. Costs and utility values for health events will be sourced from published studies including the NICE guidelines for lipid modification and hypertension. (1,35,41) Costs of medication will be sourced from the NHS National Drug Tariff.(42)

7. Change in age, treatment, CVD status and type II diabetes status

The simulation model will run for each individual for lifetime duration (death or maximum age of 100 years) and patient characteristics will be updated after each event or every 10 years (depending on which event occurs first). A 10 year update is used as the QRISK2 algorithm returns a 10 year CVD risk.

For peer review only

Table 1) Input parameters

Model inputs	Source
**1. Individual dataset**	
Population dataset	Initial patient characteristics (see Figure 2) for cohort of patients drawn from a representative national sample: Health Survey for England (HSE) dataset 2011. The dataset will include patients who meet the entry criteria for the UMPIRE trial.
**2. Calculation of baseline risks**	
Risk calculators	Risk of first CVD event and onset of type 2 diabetes estimated for individuals using QRISK2 and QDiabetes.(23, 24)<br><br>*QRISK2: 10 year CVD risk (CVD outcomes defined as angina, MI, TIA and stroke)*<br>*QDiabetes: risk of acquiring type 2 diabetes over 10 year time period*<br><br>Risks for subsequent CVD events estimated for individuals using the REACH algorithm. (38)<br>*CVD outcomes defined as cardiovascular death (includes fatal stroke and MI), non-fatal MI, non-fatal stroke, and cardiovascular hospitalisation (includes hospitalisation for unstable angina and TIA)*
Risk of heart failure	Baseline risk per age using incidence rates in Cowie et al. (1998) (43)
Relative incidence of CVD events (TIA, stroke, angina, MI)	OXVASC cohort study, Rothwell *et al.* 2005. 91,106 individuals presenting with an acute vascular event in Oxfordshire, UK in 2002-5. (34)
**3. Adherence to medication**	
Probability of adherence to treatment with usual care	Estimates from HSE 2011 dataset on adherence to relevant drugs (statins, AHT, aspirin)
Relative risk of adherence: polypill versus usual care	Estimate the probability of adherence to ≥2 AHT, statin or anti-platelet for at least four days in the preceding week for polypill group versus usual care by applying a binomial regression to the UMPIRE dataset.
**4. Treatment effects of medication (AHT, statin, anti-platelet)**	
Relative risk of CVD with treatment versus no treatment	For base case analysis, conventional meta-analysis of ITT RCT data will be used from –<br>▪ Cholesterol Treatment Trialists' Collaboration<br>▪ Blood Pressure Lowering Treatment Trialists' Collaboration<br>▪ Antithrombotic Trialists' Collaboration<br>▪ Law and Wald (44)<br>Sensitivity analysis: Test impact of adjusting for adherence within trials
**5. Other treatment outcomes (beneficial events and adverse events) and mortality rates**	
Adverse Events	
Incident type 2 diabetes	Relative risk of diabetes from statins/antihypertensives from meta-analyses of RCTs
GI bleeding	Relative risk of bleeding resulting from aspirin using estimates from meta-analyses of RCTs
Cough	Placebo-adjusted relative risk of cough resulting from ACE inhibitors using estimate from meta-analyses of RCTs

For peer review only - http://bmjopen.bmj.com/site/about/guidelines.xhtml

Reduction in heart failure	Relative risk reduction in heart failure from antihypertensives (33)
Mortality	
Stroke case fatality (60 day)<br>Age <75<br>Age > 75+	Estimate proportion of strokes that are fatal (with risks increasing with age). Estimate using the BHF Compendium of health statistics 2012, which has data from a record linkage study for England 2010.
MI case fatality (30 day)<br>Age 30-54<br>Age 55-64<br>Age 65-74<br>Age 75-84<br>Age 85+	Proportion of MI's that are fatal. Estimate using Oxford Record Linkage study.(45) National population based study, including all individuals admitted to hospital or who died suddenly from acute MI in 2010. Age was strongest predictive factor for 30-day case fatality.
Death from other causes	Estimated from national life tables (Office for National Statistics, England)(40)
**6. Costs (medication, monitoring costs, health events)**	
Drug costs (£ per year)	
Statins	National Health Service (NHS) Electronic Drug Tariff (42)
AHT drugs	
Aspirin	
Polypill	Assumed to be aggregate cost of each drug in the combined pill
Yearly monitoring costs while on medication	
Primary care nurse (£ per hour)<br>GP cost (£ per hour)<br>Lipid test (£)	Use NICE Quality Outcomes Framework to identify recommended management while on treatment (statins, AHT, antiplatelet). A cost for stopping medication will also be applied (e.g. 2 GP visits, tests as recommended in NICE clinical guidelines 181) (1)
Liver transaminase test<br>Blood tests	Costs sourced from Personal Social Services Research Unit Costs and NICE clinical guidelines 181
Costs of health states and adverse events	
Stroke	Luengo-Fernandez *et al.* 2012 (46)
TIA	
MI	NICE lipids guideline 181 (1)
Angina	
PAD	
Diabetes	
GI bleeding	
Cough (from ACE inhibitor use)	NICE lipids guidelines 127
**7. Health Related Quality of life**	
Stroke	Derived from Health Survey from England (HSE) dataset
TIA	
MI	
Angina	
PAD	
GI bleeding	
Diabetes	
Cough	

CVD: cardiovascular disease, TIA: transient ischaemic attack, MI: myocardial infarction; ITT: Intention to treat, RCT: Randomised controlled trial

For peer review only - http://bmjopen.bmj.com/site/about/guidelines.xhtml

Analysis

The simulation model will run for a sufficient number of iterations to provide stable results. Uncertainty in the model parameters will be examined using a probabilistic sensitivity analysis (PSA) which will reflect uncertainty over the values of the model inputs. Non-parametric bootstrapping of HSE data will be carried out to examine the uncertainty related to the sampling. For each PSA iteration, one non-parametric bootstrap sample will be drawn from the HSE dataset (by random sample with replacement of individuals in the dataset). An incremental analysis will be conducted and incremental cost-effectiveness ratios (ICERs) and net benefit statistics will be estimated. We will also carry out a number of sensitivity analyses to test the impact of varying uncertain parameters in the model. This will include an analysis testing the impact of varying the polypill cost.

Validation

The model will be internally and externally validated. A checklist produced by the RUPEE steering group based on current published guidelines for checking models will be used, to ensure the programmed model behaves as expected according to the theoretical model. (21, 47) The checklist includes tips for model developers, for example on the use of sensitivity analyses to test that the model is operating correctly, and re-programming complicated sections of code in another language. The model will also be reviewed and tested by an experienced modeller. The model results will be compared with real-world observations or the results of other models.

Dissemination of results

The findings of the economic evaluation will be presented to scientific and health care policy audiences in open access journals and at national and international conferences. We will also present findings to NHS policy makers and pharmaceutical companies.

DISCUSSION

Medication adherence is important for disease management, and benefits of increased adherence to preventative medication for CVD include improved clinical outcomes. (5) The UMPIRE clinical trial was conducted to evaluate the effect of a polypill strategy compared to usual care on adherence. It showed that the polypill strategy significantly augmented adherence and this was reflected by improvements in SBP and LDL-C. (8) Whether or not this impact remains in the long term cannot be determined from the trial data alone. The RUPEE (NHS) study is being conducted to evaluate the long term impact of a polypill strategy; in particular, the analysis will evaluate the long term impact of increased adherence on outcomes. An economic model is being developed to estimate the long term costs and QALYs associated with implementing a polypill strategy in the NHS compared to usual

For peer review only - http://bmjopen.bmj.com/site/about/guidelines.xhtml

care. This analysis will represent the first comprehensive cost effectiveness analysis using directly applicable clinical trial data.

This paper outlines the process behind the design of the economic model. We carried out a review of published CVD models to identify a modelling approach that would suit the health care decision: use of a polypill versus usual care in a population with or at high risk of CVD. We identified an individual simulation model as the most appropriate approach as it allows the heterogeneity in the population to be adequately reflected. The model will use validated disease risk algorithms to estimate the probability of an individual experiencing a CVD event or the onset of diabetes. Individuals can also experience an increased risk of an adverse event (diabetes, cough and gastrointestinal bleeding) from treatment. The risk of a CVD event will be reduced if the individual is adherent to treatment. We will simulate adherence to treatment using data from the HSE 2011 dataset with an increased relative risk reduction if the person is adherent to the polypill. The relative risk of adherence to the polypill versus usual care will be sourced from the UMPIRE trial data for the English population. Costs and QALYs will be estimated for each individual and aggregated across the sample population (based on the HSE 2011 dataset).

The RUPEE (NHS) model will have a number of advantages over existing models constructed to evaluate a CVD polypill. (48-50) One advantage is the use of an individual simulation model which will allow us to capture the heterogeneity in the variation in CVD risk in the UK population unlike other models which use Markov type transition state models. Another is that we will extrapolate data on adherence to medication from a nationally representative population dataset (Health Survey for England) which will allow us to simulate adherence per individual rather than assuming a constant adherence across our population. We will also allow for adverse events from treatment and treatment cessation, therefore more accurately reflecting clinical practice.

It would be preferable to use per protocol treatment effectiveness data in our analysis as intention-to-treat data already accounts for adherence (people switching and ceasing medication during the trial period). However, per protocol data is difficult to obtain for all drugs, therefore we will use the ITT treatment effect data and carry out sensitivity analyses to test the impact.

The introduction of a CVD preventive polypill strategy will simplify pill taking for patients potentially leading to greater adherence and better health outcomes. This analysis will provide information on the cost-effectiveness of the polypill in a NHS setting.

For peer review only - http://bmjopen.bmj.com/site/about/guidelines.xhtml

Contributors: CC carried out the literature review and drafted the manuscript. JL, ST, HMD, HW and NP, AR and SJ contributed to the development of the protocol. JL provided input on the heath economics model. HMD provided statistical advice and ST, HW and NP contributed clinical advice. AR and SJ peer reviewed draft manuscripts and contributed to the final version of the protocol. All authors approved the final version of the manuscript submitted for publication.

Funding: This work was supported by the National Institute for Health Research (NIHR), grant number [grant 241849]. UMPIRE was funded by the European Commission Framework Program 7.

Competing interests: ST, NP and HW acknowledge support from the NIHR Biomedical Research Centre at Imperial College Healthcare NHS Trust and Imperial College London. SJ and AR are employed by The George Institute for Global Health who own the IP for the polypill in Australia.

Ethics approval: No ethics approval was required

This paper presents independent research funded by the National Institute for Health Research (NIHR) under its Research for Patient Benefit (RfPB) Programme (Grant Reference Number PB-PG-1112-29080). The views expressed are those of the author(s) and not necessarily those of the NHS, the NIHR or the Department of Health.

For peer review only

For peer review only - http://bmjopen.bmj.com/site/about/guidelines.xhtml

References

(1) National Institute for Health and Care Excellence (NICE*)*,. Lipid modification: cardiovascular risk assessment and the modification of blood lipids for the primary and secondary prevention of cardiovascular disease. 2014;Clinical Guideline 181.

(2) JBS3 Board. Joint British Societies' consensus recommendations for the prevention of cardiovascular disease (JBS3). *Heart* 2014 Apr;100 Suppl 2:ii1-ii67.

(3) Yusuf S, Islam S, Chow CK, et al. Use of secondary prevention drugs for cardiovascular disease in the community in high-income, middle-income, and low-income countries (the PURE Study): a prospective epidemiological survey. *The Lancet* 2011;378(9798):1231-1243.

(4) Osterberg L, Blaschke T. Adherence to medication. *N Engl J Med* 2005;353(5):487-497.

(5) Simpson RJ, Mendys P. The effects of adherence and persistence on clinical outcomes in patients treated with statins: a systematic review. *Journal of clinical lipidology* 2010;4(6):462-471.

(6) Connor J, Rafter N, Rodgers A. Do fixed-dose combination pills or unit-of-use packaging improve adherence? A systematic review. *Bull World Health Organ* 2004;82(12):935-939.

(7) Elley CR, Gupta AK, Webster R, et al. The efficacy and tolerability of 'polypills': meta-analysis of randomised controlled trials. *PLoS One* 2012;7(12):e52145. doi: 10.1371/journal.pone.0052145

(8) Thom S, Field J, Poulter N, et al. Use of a Multidrug Pill In Reducing cardiovascular Events (UMPIRE): rationale and design of a randomised controlled trial of a cardiovascular preventive polypill-based strategy in India and Europe. *Eur.J.Prev.Cardiol*. 2014; 21(2) 252-261

(9) Thom S, Poulter N, Field J, et al. Effects of a fixed-dose combination strategy on adherence and risk factors in patients with or at high risk of CVD: the umpire randomized clinical trial. *JAMA* 2013;310(9):918-929.

(10) Caro JJ, Briggs AH, Siebert U, et al. ISPOR-SMDM Modeling Good Research Practices Task Force. Modeling good research practices--overview: a report of the ISPOR-SMDM Modeling Good Research Practices Task Force-1. *Med Decis Making* 2012 Sep-Oct;32(5):667-677.

(11) Roberts M, Russell LB, Paltiel AD, et al. Conceptualizing a model: a report of the ISPOR-SMDM Modeling Good Research Practices Task Force-2. *Med Decis Making* 2012 Sep-Oct;32(5):678-689.

(12) National Institute for Health and Care Excellence (NICE),. Guide to the methods of technology appraisal 2013.

(13) Barton P, Andronis L, Briggs A,et al. Effectiveness and cost effectiveness of cardiovascular disease prevention in whole populations: modelling study. *BMJ* 2011 Jul 28;343:d4044.

(14) Weinstein MC, Coxson PG, Williams LW,et al. Forecasting coronary heart disease incidence, mortality, and cost: the Coronary Heart Disease Policy Model. *Am J Public Health* 1987 Nov;77(11):1417-1426.

For peer review only

For peer review only - http://bmjopen.bmj.com/site/about/guidelines.xhtml

(15) Cooper K, Davies R, Roderick P, et al. The development of a simulation model of the treatment of coronary heart disease. *Health Care Manag Sci* 2002;5(4):259-267.

(16) Whitfield MD, Gillett M, Holmes M, et al. Predicting the impact of population level risk reduction in cardio-vascular disease and stroke on acute hospital admission rates over a 5 year period—a pilot study. *Public Health* 2006;120(12):1140-1148.

(17) Unal B, Capewell S, Critchley JA. Coronary heart disease policy models: a systematic review. *BMC Public Health* 2006 Aug 18;6:213.

(18) Capewell S, Allender S, Critchley J, et al. Modelling the UK burden of cardiovascular disease to 2020: a research report for the Cardio & Vascular Coalition and the British Heart Foundation. *British Heart Foundation* 2008(Available at http://www.healthimpact.org.uk/content/resources/z154_modelling_the_burden_of_cv_disease.pdf).

(19) Hotchkiss JW, Davies C, Gray L, et al. Trends in adult cardiovascular disease risk factors and their socio-economic patterning in the Scottish population 1995-2008: cross-sectional surveys. *BMJ Open* 2011 Aug 9;1(1):e000176-2011-000176.

(20) Ward S, Jones ML, Pandor A, et al. A systematic review and economic evaluation of statins for the prevention of coronary events. 2007. Health Technology Assessment 2007; 11(14):1-160, iii-iv.

(21) Davis S, Stevenson M, Tappenden P, et al. NICE DSU Technical Support Document 15: Cost-effectiveness modelling using patient level simulation. 2014.

(22) Hippisley-Cox J, Coupland C, Vinogradova Y, et al. Predicting cardiovascular risk in England and Wales: prospective derivation and validation of QRISK2. *BMJ* 2008 Jun 28;336(7659):1475-1482.

(23) Hippisley-Cox J, Coupland C, Robson J, et al. Predicting risk of type 2 diabetes in England and Wales: prospective derivation and validation of QDScore. *BMJ* 2009 Mar 17;338:b880.

(24) Hippisley-Cox J, Coupland C. Unintended effects of statins in men and women in England and Wales: population based cohort study using the QResearch database. *BMJ*2010;340.

(25) Macedo AF, Taylor FC, Casas JP, et al. Unintended effects of statins from observational studies in the general population: systematic review and meta-analysis. *BMC Med* 2014 Mar 22;12:51-7015-12-51.

(26) Elliott WJ, Meyer PM. Incident diabetes in clinical trials of antihypertensive drugs: a network meta-analysis. *The Lancet* 2007;369(9557):201-207.

(27) Gupta AK, Dahlof B, Dobson J, et al. Determinants of new-onset diabetes among 19,257 hypertensive patients randomized in the Anglo-Scandinavian Cardiac Outcomes Trial--Blood Pressure Lowering Arm and the relative influence of antihypertensive medication. *Diabetes Care* 2008 May;31(5):982-988.

(28) Antithrombotic Trialists' Collaboration. Collaborative meta-analysis of randomised trials of antiplatelet therapy for prevention of death, myocardial infarction, and stroke in high risk patients. *BMJ* 2002 Jan 12;324(7329):71-86.

For peer review only - http://bmjopen.bmj.com/site/about/guidelines.xhtml

(29) Baigent C, Blackwell L, Collins R, et al. Antithrombotic Trialists'(ATT) Collaboration. Aspirin in the primary and secondary prevention of vascular disease: collaborative meta-analysis of individual participant data from randomised trials. *The Lancet* 2009;373(9678):1849-1860.

(30) Peters R, Beckett N, Burch L, et al. The effect of treatment based on a diuretic (indapamide) ± ACE inhibitor (perindopril) on fractures in the hypertension in the very elderly trial (HYVET). *Age Ageing* 2010;39(5):609–16.

(31) SPRINT. A Randomized Trial of Intensive versus Standard Blood-Pressure Control. *N Engl J Med* 2015; 373:2103-2116

(32) Finegold J A, Manisty CH, Goldacre B, et al. What proportion of symptomatic side effects in patients taking statins are genuinely caused by the drug? Systematic review of randomized placebo-controlled trials to aid individual patient choice. *Eur J Prev Cardiol*. 2014; 21: 464

(33) Ettehad D, Emdin CA, Kiran A, et al. Blood pressure lowering for prevention of cardiovascular disease and death: A systematic review and meta-analysis. *The Lancet.* 2015;387(10022):957–67

(34) Rothwell PM, Fowkes FGR, Belch JFF, et al. Effect of daily aspirin on long-term risk of death due to cancer: analysis of individual patient data from randomised trials. *The Lancet.* 2011; 377(9759):31-

(35) National Institute for Health and Care Excellence (NICE),. Lipid Modification Cardiovascular risk assessment and the modification of blood lipids for the primary and secondary prevention of cardiovascular disease. *NICE Clinical Guidelines* 67 2008 (Modified 2010).

(36) D'Agostino RB, Vasan RS, Pencina MJ, et al. General cardiovascular risk profile for use in primary care the Framingham Heart Study. *Circulation* 2008;117(6):743-753.

(37) Collins GS, Altman DG. An independent and external validation of QRISK2 cardiovascular disease risk score: a prospective open cohort study. *BMJ* 2010;340.

(38) Wilson PW, D'Agostino R, Bhatt DL, et al. An international model to predict recurrent cardiovascular disease. *Am J Med* 2012;125(7):695-703. e1.

(39) Rothwell P, Coull A, Silver L, et al. Population-based study of event-rate, incidence, case fatality, and mortality for all acute vascular events in all arterial territories (Oxford Vascular Study). *The Lancet* 2005;366(9499):1773-1783.

(40) Office for National Statistics. Available at: http://www.ons.gov.uk/ons/taxonomy/index.html?nscl=Life+Tables.

(41) National Institute for Health and Clinical Excellence,. Hypertension: clinical management of primary hypertension in adults. 2011;*Clinical Guideline 127*.

(42) National Health Services (NHS) Business Services Authority Drug Tariff. Available at: http://www.nhsbsa.nhs.uk/PrescriptionServices/4940.aspx.

For peer review only - http://bmjopen.bmj.com/site/about/guidelines.xhtml

(43) Cowie MR WDCATSP-WPSVaSG. Incidence and aetiology of heart failure. A population based study. European Heart Journal. 1999; 20: 421-428

(44) Law M, Morris J, Wald N. Use of blood pressure lowering drugs in the prevention of cardiovascular disease: meta-analysis of 147 randomised trials in the context of expectations from prospective epidemiological studies. *BMJ* 2009;338. 37

(45) Smolina K, Wright FL, Rayner M, et al. Determinants of the decline in mortality from acute myocardial infarction in England between 2002 and 2010: linked national database study. *BMJ* 2012;344. 38

(46) Luengo-Fernandez R, Gray AM, Rothwell PM. A Population-Based Study of Hospital Care Costs During 5 Years After Transient Ischemic Attack and Stroke. *Stroke* 2012;43(12):3343-3351. 39

(47) Philips Z, Bojke L, Sculpher M, et al. Good practice guidelines for decision-analytic modelling in health technology assessment. *Pharmacoeconomics* 2006;24(4):355-371.

(48) Becerra V, Gracia A, Desai K, et al. Cost-effectiveness and public health benefit of secondary cardiovascular disease prevention from improved adherence using a polypill in the UK. *BMJ Open* 2015 May 9;5(5):e007111-2014-007111.

(49) Newman J, Grobman WA, Greenland P. Combination Polypharmacy for Cardiovascular Disease Prevention in Men: A Decision Analysis and Cost-Effectiveness Model. *Preventive cardiology* 2008;11(1):36-41.

(50) van Gils PF, Over EAB, Hamberg-van Reenen HH, et al. The polypill in the primary prevention of cardiovascular disease: cost-effectiveness in the Dutch population. *BMJ Open* 2011;1:e000363 doi:101136/bmjopen-2011-000363

For peer review only

For peer review only - http://bmjopen.bmj.com/site/about/guidelines.xhtml

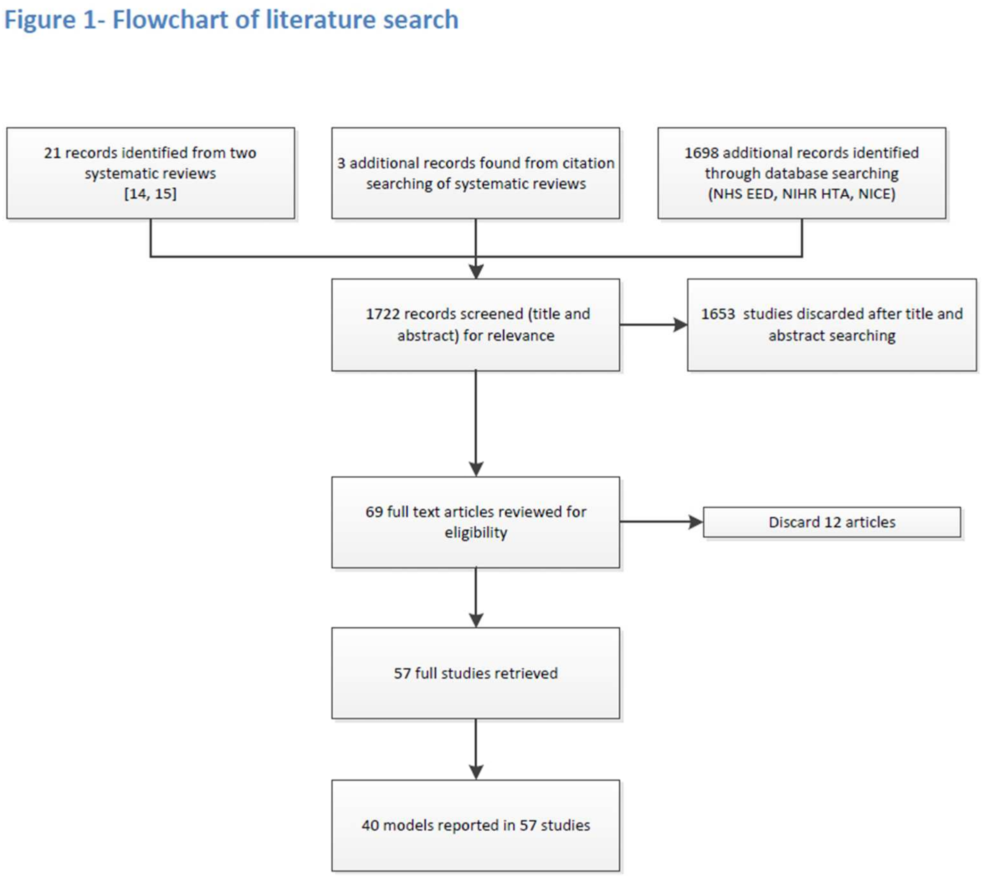

Flowchart of literature review

232x206mm (96 x 96 DPI)

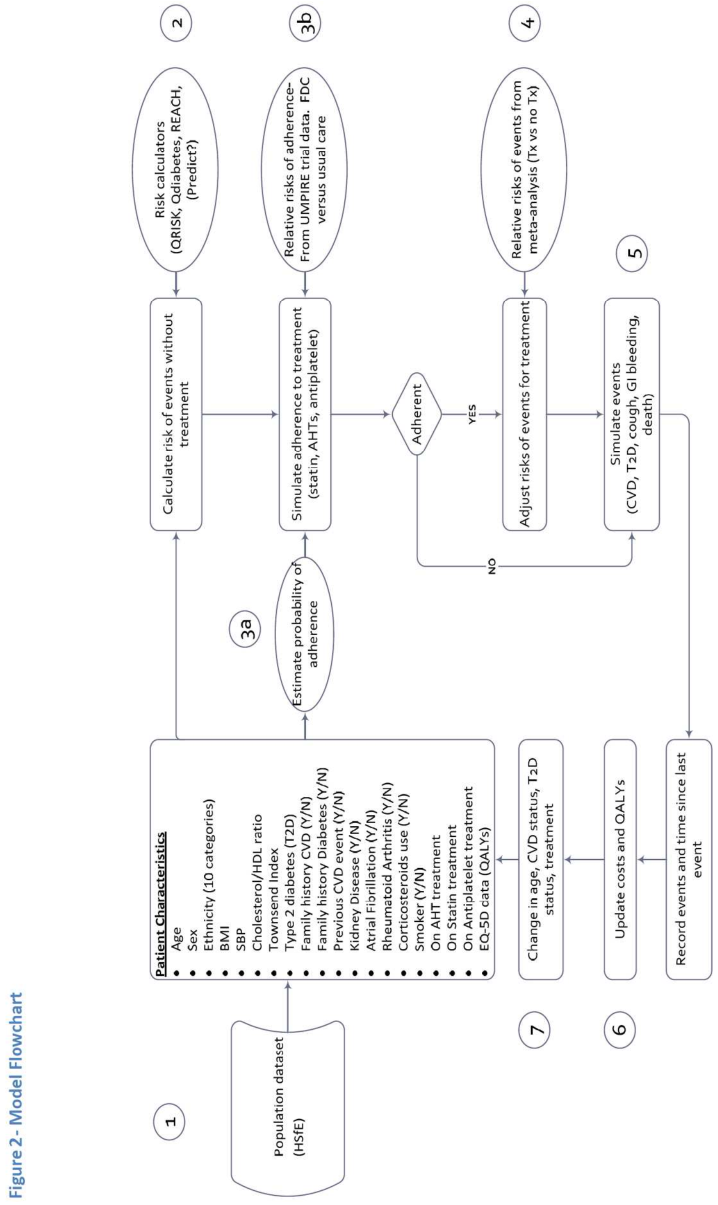

Model Figure Simulation

417x702mm (96 x 96 DPI)

A Protocol for an economic evaluation of a polypill in patients with established or at high risk of cardiovascular disease in a UK NHS setting: RUPEE (NHS) study

Journal:	*BMJ Open*
Manuscript ID	bmjopen-2016-013063.R1
Article Type:	Protocol
Date Submitted by the Author:	17-Aug-2016
Complete List of Authors:	Crossan, Catriona; Health Economics Research Group<br>Dehbi, Hakim-Moulay; Imperial College London, Department of Epidemiology and Biostatistics<br>Thom, Simon; Imperial College London, International Centre for Circulatory Health<br>Poulter, Neil; Imperial College London<br>Williams, Hilarie; Imperial College London, International Centre for Circulatory Health<br>Rodgers, Anthony; The George Institute for Global health,<br>Jan, Stephen; The George Institute for Global Health,<br>Lord, Joanne; University of Southampton, Health Technology Assessments Centre
**Primary Subject Heading**:	Health economics
Secondary Subject Heading:	Cardiovascular medicine, Patient-centred medicine
Keywords:	Coronary heart disease < CARDIOLOGY, HEALTH ECONOMICS, Coronary intervention < CARDIOLOGY

SCHOLARONE™ Manuscripts

A Protocol for an economic evaluation of a polypill in patients with established or at high risk of cardiovascular disease in a UK NHS setting: *RUPEE (NHS) study*

Authors: Crossan C, Dehbi HM, Thom S, Poulter N, Williams H, Rodgers A, Jan S, Lord J

Corresponding author: Catriona Crossan, Health Economics Research Group, Brunel University London, Uxbridge, Middlesex, UB8 3PH, United Kingdom. catriona.crossan@brunel.ac.uk Tel: 0044 (0) 1895 267920. Fax:0044 (0) 1895 269708

Author Affiliations

Hakim-Moulay Dehbi. Department of Epidemiology and Biostatistics, Imperial College, London, W2 1PG, United Kingdom.

Simon Thom. Peart-Rose Research Unit, International Centre for Circulatory Health NHLI, Imperial College London (Hammersmith Campus), Du Cane Road, London, W12 0HS, United Kingdom.

Neil Poulter. Peart-Rose Research Unit, International Centre for Circulatory Health NHLI, Imperial College London (Hammersmith Campus), Du Cane Road, London, W12 0HS, United Kingdom.

Hilarie Williams. Peart-Rose Research Unit, International Centre for Circulatory Health NHLI, Imperial College London (Hammersmith Campus), Du Cane Road, London, W12 0HS, United Kingdom.

Anthony Rodgers. The George Institute for Global Health, University of Sydney, Camperdown, New South Wales, Australia

Stephen Jan. The George Institute for Global Health, University of Sydney, Camperdown, New South Wales, Australia

Joanne Lord. Southampton Health Technology Assessments Centre, University of Southampton, 1st Floor, Epsilon House, Enterprise Road, Southampton Science Park, Southampton SO16 7NS

Keywords: Cardiovascular disease, polypill, adherence, cost-effectiveness, economic evaluation

Word Count 4,951

Figures 2 **Tables** 1

For peer review only

Abstract

Introduction: The 'Use of a Multi-drug Pill in Reducing cardiovascular Events' (UMPIRE) trial was a randomised controlled clinical trial evaluating the impact of a polypill strategy on adherence to indicated medication in a population with established cardiovascular disease of or at high risk thereof. The aim of RUPEE-NHS is to estimate the potential health economic impact of a polypill strategy for CVD prevention within the NHS using UMPIRE trial and other relevant data. This paper describes the design of a modelled economic evaluation of the impact of increased adherence to the polypill versus usual care amongst the UK UMPIRE participants.

Methods and Analysis: As recommended by ISPOR-SMDM modelling guidelines a review of published CVD models was undertaken to identify the most appropriate modelling approach and structure. The review was carried out in the electronic databases, MEDLINE and EMBASE. 40 CVD models were identified from 57 studies, the majority of economic models were health state transition cohort models and individual level simulation models. The findings were discussed with clinical experts to confirm the approach and structure. An individual simulation approach was identified as the most suitable method to capture the heterogeneity in population CVD risk. RUPEE-NHS will use UMPIRE trial data on adherence to estimate the long term cost-effectiveness of the polypill strategy.

Dissemination: The evaluation findings will be presented in open access scientific and healthcare policy journals and at national and international conferences. We will also present findings to NHS policy makers and pharmaceutical companies.

Strengths and Limitations

This paper provides a clear outline of how a model for an economic evaluation is developed.

Providing an outline of the model structure which includes details on the underlying epidemiology and data inputs will add transparency to the findings of the RUPEE-NHS study

Though the model has been designed to include all major adverse and beneficial effects of treatment, the model structure will not include every potential treatment effect, for example the benefits of treatment on Alzheimer's disease will not be included.

For peer review only - http://bmjopen.bmj.com/site/about/guidelines.xhtml

INTRODUCTION

Adherence to recommended preventive medication regimes (1,2) in people at high risk of cardiovascular disease (CVD) is low, even in high income countries. (3) Poor adherence is associated with greater deterioration in health status and increased health care costs (4) and studies have shown that improved adherence to medication is associated with clinical benefits.(5) CVD preventive medication typically involves several drugs and adherence is inversely proportional to the number of prescriptions. Furthermore, physician inertia and patient resistance present barriers to initiating or restarting full recommended therapy. A single pill that includes several indicated drugs (a "polypill"), may improve long-term adherence by addressing these issues. If the polypill is priced lower than the price of the pills bought separately, it will also make it more affordable. (6,7) The UMPIRE (Use of a Multidrug Pill in Reducing Cardiovascular events) clinical trial was set up to evaluate the polypill in patients with or at high risk of CVD.

The UMPIRE trial randomised 2004 participants with established CVD (prior CVD event such as stroke or myocardial infarction) or at high risk of CVD (defined as a 5 year risk of >15%) based in India, England, Ireland and The Netherlands to either the polypill or usual care. The primary outcome of the trial was adherence to indicated treatments (statin, aspirin and two blood pressure lowering drugs), measured as self-reported current use of antiplatelet, statin and ≥2 blood pressure lowering therapies for at least 4 days in the week preceding visits (baseline and end of trial visits). Other outcomes included systolic blood pressure (SBP) and low-density lipoprotein cholesterol (LDL-C). The trial found that the use of a polypill strategy resulted in greater adherence to treatment at 15 months and significant improvements in SBP and LDL-C. Detailed results and a description of the UMPIRE trial protocol are available. (8, 9)

UMPIRE collected data on resource use and self-reported health related quality of life using the EQ-5D. In order to estimate the long term costs and health outcomes associated with the polypill strategy an economic model is required. Due to differences in the patient population, care pathway and health care costs, separate analyses are needed for the four participating countries.

The analysis of the UMPIRE English trial data, (Researching the UMPIRE Processes for Economic Evaluation in the National Health Service (RUPEE-NHS)), aims to estimate the cost-effectiveness of the polypill strategy compared to conventional multi-drug therapy for the prevention of established cardiovascular disease in English NHS patients with or at high risk of CVD. The RUPEE (NHS) study

For peer review only - http://bmjopen.bmj.com/site/about/guidelines.xhtml

will use UMPIRE English trial data on adherence to the polypill and will develop an economic model to estimate cost effectiveness.

The aim of this paper is to detail the modelling plan for the RUPEE (NHS) study.

METHODS

Model design process

An economic model has been described as a mathematical framework that represents reality at an adequate level of detail to inform clinical or policy decisions. (10) Guidelines on modelling produced by the International Society for Pharmacoeconomics and Outcomes Research (ISPOR) and the Society for Medical Decision Making (SMDM) joint taskforce recommend that it is best practice to carry out a conceptualisation process prior to programming the economic model. This process has two distinct components: specification of the study question and economic model. (11)

Specification of the study question

The first component informs choices about how to structure the economic model and parameters. The RUPEE (NHS) study aims to evaluate two different treatment strategies in a population with or at high risk of CVD. The population for the economic model is defined by the inclusion criteria of the UMPIRE trial. (9) The inclusion criteria are listed below:

- Aged ≥18 years and
- High CVD risk defined as either established atherothrombotic CVD (history of coronary heart disease (CHD), ischaemic cerebrovascular disease, or peripheral arterial disease (PAD)) or a 5 year risk of ≥15% calculated using the Framingham risk equation

The economic model will evaluate the polypill strategy compared to usual medication. In the UMPIRE trial, participants assigned to the polypill received one of 2 versions: version 1 contained aspirin 75mg, simvastatin 40mg, lisinopril 10mg and atenolol 50mg, and version 2 contained the same ingredients but substituted hydrochlorothiazide 12.5mg for atenolol 50mg. Participants assigned to usual care continued taking medications as prescribed by their general practitioner (GP).

The RUPEE (NHS) study will follow guidelines for modelling health technologies as recommended by the National Institute for Health and Care Excellence. (NICE) (12) Therefore a NHS and Personal Social Services (PSS) perspective will be adopted to measure health service resource use and health related quality of life will be measured by quality adjusted life years (QALYs) obtained using the EQ-

For peer review only - http://bmjopen.bmj.com/site/about/guidelines.xhtml

5D. As per the NICE guidelines, costs and QALYS will be discounted at a rate of 3.5% per year. (12) The time horizon reflected in the economic model will be lifetime to represent the chronic nature of CVD.

Conceptualisation of the economic model

The second component of the conceptualisation process involves defining the economic model. There are two steps to this approach. The first step is to identify the appropriate modelling approach. The modelling approach defines the analytical framework of the economic model. Different types of analytical frameworks have been used to represent CVD including decision trees, state transition models, compartmental models, individual simulation models and hybrid models which often combine elements from different frameworks. (13-17)

The second step determines the underlying structure of the analytical framework, which will represent the disease and care pathway. The modelling approach needs to reflect: 1) CVD disease and care pathway for this population; 2) the beneficial and adverse effect of treatment (polypill or usual care); 3) the impact of increased adherence to treatment on health outcomes.

The guidelines produced by ISPOR-SMDM on modelling recommend that existing models addressing related problems should be reviewed as this approach can help identify both the modelling approach and underlying structure.(11) To inform the RUPEE (NHS) economic model, we carried out a review of published models evaluating interventions for CVD.

Review of published CVD economic models

The purpose of the literature review was to identify the appropriate analytical framework to represent the decision problem. The literature review also aimed to inform the underlying model structure: disease and care pathway.

Search strategy

The search strategy was conducted using the NHS Economic Evaluation Database (NHS EED), the NIHR Health Technology Assessment (HTA) monograph series and the NICE guidelines website. The search terms used included 'cardiovascular disease', 'coronary heart disease', 'stroke', 'myocardial infarction', 'angina' and 'peripheral artery disease'. Studies were excluded from the review if they did not discuss the development or review of an economic model; if no disease states for cardiovascular disease were included in the model; if the focus of the study was a diagnostic test or surgical intervention where the economic model used a time frame of <10 years. Studies were not excluded on the basis of intervention (drug treatment or lifestyle intervention) or on the basis of

For peer review only - http://bmjopen.bmj.com/site/about/guidelines.xhtml

For peer review only

date published or language. We developed a data extraction form which included fields on model purpose, structure, health states and events, transparency and validation. We did not collate information about the findings of the model as the objective of the review was to identify alternative model frameworks and methods used to represent CVD.

An initial general literature search identified a 2006 systematic review of CHD policy models by Unal *et al*. which was updated in 2008 by Capewell *et al*. and expanded to include stroke models. (17,18) The review by Capewell *et al*. identified seven 'notable' CHD models (of which six had been identified in the previous review by Unal *et al.*), nine stroke models and several models that were currently in development at the time of publication. We reviewed the notable models and models in development identified by Capewell *et al*. Citation searching of both systematic reviews was carried out to identify other models published since 2008.

Review findings

Overall 57 studies were identified which reported on 40 CVD models. Figure 1 presents the flowchart for the search strategy.

The search found several studies which reported on the same model, for example the IMPACT CHD model developed by Capewell *et al*. was used in analyses of CVD in other populations. (19) In some cases, a model was adapted for different analyses, such as the Sheffield model which was developed to evaluate statin therapy and was then adapted for use in the development of the NICE guidelines for lipid modification. (1,20) The Sheffield model was also partially used in a whole population modelling study by Barton *et al*. (13)

Further details on the review can be found in the supplementary appendix. The appendix includes a list of the reviewed models (see Table 1 supplementary appendix), an example of the data extraction form and an example of an illustration and details of one of the reviewed models (see Figure 1 supplementary appendix). Schematic illustrations of several models were used in discussions with clinical experts about the different types of modelling approaches

[Figure 1- Flowchart for search strategy for CVD models]

Modelling approach

The search identified that the two most commonly used modelling approaches were health state transition cohort models and individual-level simulation models. Both approaches were critically assessed to determine their suitability to capture the disease and care pathway.

For peer review only - http://bmjopen.bmj.com/site/about/guidelines.xhtml

A cohort model can be defined as any model which estimates the outcomes for a group of patients, whereas with a patient level simulation, outcomes are evaluated at the individual level. Therefore, one of the main differences between the two approaches is how they estimate costs and QALYs: cohort models estimate expected costs and QALYs for the modelled population as a whole, whereas individual level simulation models estimate cost and QALYs for each individual and the average is taken across the sample.

With a health state transition cohort model, the population progresses through a set of mutually exclusive health states at regular intervals called cycles, determined by a predefined transition matrix. Health state transition cohort models are also commonly called Markov models. However, such models are only Markovian when they display the Markovian 'memoryless' property where the progression of the patient through the model is only dependent on the current state in which the patient resides and not on anything that happened before they entered that health state. It is also possible to model at the individual level using a state transition model by sampling probabilities for each individual patient to experience a particular transition in each model cycle. (21)

Both model approaches can use a discrete time approach: with this approach the model cycle length will be defined in advance. The cohort or individual progress through health states or events which represent the disease pathway and only one event may occur within each cycle length. Costs and QALYs are updated once per cycle. Alternatively, individual level simulation models are often set up as discrete event simulations (DES). With a DES approach, an event can occur at any time point, for example, an event could occur at three months, one year and twenty years. As an event occurs, costs and QALYs are recorded and updated for each individual.

A health state transition model was used to develop NICE guidance for lipid modification treatment. (1) The limitation of this approach is that it may be unable to capture the underlying heterogeneity in the population. Individual CVD risk can be estimated using CVD risk algorithms such as QRISK2 which use a range of patient characteristics such as age, sex, ethnicity, systolic blood pressure and body mass index to estimate a 10 year CVD risk.(22) To capture this complexity in a health state transition model would require the construction of a large number of subgroups to reflect different subsets of patient characteristics and the variation in CVD risk in the population. This could become impractical to model. It also has the disadvantage that accuracy could be lost by using representative values for subgroups. An individual simulation model structure may be more

For peer review only - http://bmjopen.bmj.com/site/about/guidelines.xhtml

appropriate to model the level of detail required to estimate CVD outcomes reflective of those in the population.

The Markovian memoryless property means that data on individual patients' history is not retained as they progress through the model. Accounting for individual patient history in a Markov model would require multiplying the number of health states to an infeasible level where the model would become too complex and impracticable to run.

To accurately identify the effectiveness of each treatment strategy in a population with or at high risk of CVD, an individual simulation model was deemed the most appropriate for the RUPEE (NHS) study to reflect the heterogeneity in the population which impacts on the risk of a CVD event and subsequent costs and outcomes. The individual simulation model will use a discrete event approach to handle time.(21)

Model structure

The findings of the review were discussed with clinical experts to confirm the health events and the methods used to model the progression of persons through the disease pathway.

Model events (CVD, diabetes and adverse events)

The most commonly included types of CVD events in the reviewed models were CHD (angina and myocardial infarction), cerebrovascular events (transient ischaemic attack (TIA) and stroke) and peripheral arterial disease (PAD). It was decided that the CVD events relevant for the current model would reflect those most commonly included in prior such models. PAD will not be included as a CVD event in the model as there is less likely to be a definable acute PAD event compared to other CVD events such as MI and stroke. We will assume that patients can experience more than one CVD event in their lifetime. The risk of CVD will also be assumed to change with age in the model.

Diabetes is a risk factor for CVD with a substantial cost and impact on health related quality of life, therefore diabetes will be included as a comorbidity in the model. The risk of new onset diabetes will be estimated using the QDiabetes risk algorithm.(23)

Adverse effects from treatment will include an increase in the risk of new onset diabetes resulting from treatment with statins and antihypertensive drugs. (24-27) The risk of a persistent cough resulting from treatment with angiotensin-converting-enzyme inhibitors (ACE inhibitors) will be included as an event. The probability of a cough resulting from treatment will be sourced from

For peer review only - http://bmjopen.bmj.com/site/about/guidelines.xhtml

meta-analyses of randomised controlled trial (RCT) data for ACE inhibitors. As aspirin use is associated with an increased risk of gastrointestinal bleeding,(28,29) an increased risk of gastrointestinal bleeding from treatment with aspirin will be included.

Renal impairment will not be included in the model as an adverse effect of ACE inhibitors. Whilst ACE inhibitors may cause an acute rise in serum creatinine in a few patients with renal artery stenosis and more generally cause a slight short term increase in creatinine levels, the effects are complex and there may be a net improvement in renal function overall in a treated population. The rate of falls and fractures will be estimated not to alter, given the evidence from randomised trials of blood pressure lowering agents, although this is an area of debate with regard to patients with higher levels of frailty. (30, 31)

Other adverse effects from statin treatment such as liver dysfunction and myopathy will not be included in the model as these cases are rare and are assumed to have a minimal impact on outcomes. (1) (32)

Treatment with antihypertensives is associated with a reduction in heart failure, therefore this will be included as an outcome in the model. (33) Other outcomes of treatment are likely but will not be included – for example a reduction in cancer with aspirin use of more than 5 years. (34)

Progression of individuals through model

The progression of persons through the disease pathway differs depending on the modelling approach: health state transition models such as the Markov model developed for NICE guidelines on lipid modification use a predefined transition matrix to determine progression through the CVD health states.(35) Alternatively, simulation models can use risk algorithms to estimate the probability of CVD events or new onset diabetes. The NICE guidelines for lipid modification recommend the use of QRISK2, which is a risk algorithm derived to estimate primary CVD risk in UK populations. (1,22) The QRISK2 risk algorithm predicts the risk of a 10 year CHD event (angina, MI) or a cerebrovascular event (TIA, stroke). It does not include the risk of PAD. An alternative CVD risk algorithm is the Framingham equation;(36), however, a validation study comparing QRISK2 and Framingham found that QRISK2 is better calibrated to a UK population.(37) The RUPEE (NHS) model will therefore use the QRISK2 risk algorithm.

For peer review only - http://bmjopen.bmj.com/site/about/guidelines.xhtml

RUPEE (NHS) economic model

Figure 2 depicts the flowchart of the RUPEE (NHS) model structure. The oval shapes represent data inputs to the model, whereas the rectangular shapes represent processes.

[Figure 2 - Flowchart of RUPEE (NHS) model structure]

Model description

In the RUPEE (NHS) model costs and QALYs are recorded for each individual and an average cost and QALY for the simulated population are estimated. The RUPEE (NHS) model will be run twice, once to simulate costs and QALYs under usual care and once to simulate costs and QALYs under the polypill scenario (polypill scenario will include polypill version 1 and version 2). Individuals representing the UMPIRE trial inclusion criteria will enter the model (label 1 in Figure 2), and their baseline risk of a CVD event and onset diabetes will be estimated using the QRISK2 CVD risk algorithm and QDiabetes algorithm (label 2 in Figure 2) respectively. For each individual, whether or not they are adherent to medication will be simulated using Monte Carlo simulation based on the probability of adherence in usual care (label 3a in Figure 2). If the individual is simulated to be adherent to medication their risk of a CVD event will be modified by a treatment effect (label 4 in Figure 2). In the polypill scenario of the model, the probability of adherence will be further modified by the relative risk of adherence to medication. The relative risk of adherence to medication will be sourced from the UMPIRE trial data (label 3b in Figure 2). Individuals may experience a CVD event or onset of diabetes based on their estimated CVD and diabetes risk, which will be estimated using the QRISK2 and QDiabetes algorithms. Individuals may also experience an adverse reaction to medication (if adherent) including gastrointestinal bleeding, early onset of diabetes and a persistent cough. Costs and QALYs will be recorded for each event (including adverse events). Individuals can experience more than one event (model run for lifetime horizon) and patient characteristics such as age and history of previous events, such as a stroke or new onset diabetes, are updated during the model run, with an ensuing reflective increase in the risk of an event.

Input parameters

Each point in the flowchart is labelled and a description of the process or data requirement label is described below. Table 1 provides further details on data input parameters for the RUPEE-NHS model and potential sources of data.

1. Population Dataset

We will use the 2011 Health Survey for England (HSE) as a population dataset for the economic model. The HSE is a cross sectional survey which contains anonymised information on a representative sample of the population. The 2011 HSE dataset collected information on CVD,

For peer review only - http://bmjopen.bmj.com/site/about/guidelines.xhtml

including individual CVD events and medication history. The dataset also contains information on demographic and socio-economic characteristics and health related data such as body mass index (BMI), SBP and LDL-C and history of CVD events. These data are required in order to estimate individual baseline risks of CVD and diabetes in the model.

2. Calculation of baseline risks of events without treatment

Baseline risks for CVD for each sampled individual will be calculated using published risk algorithms. As per recent NICE guidance for lipid modification, we will use the recommended algorithm for CVD risk, QRISK2. (1,22) The algorithm was derived using QRESEARCH, a large database derived from the pseudonymised health records of over 13 million patients registered with a general practitioner in the UK. If an individual has established CVD (previously experienced a CVD event), we will estimate a secondary CVD risk using the REACH algorithm. (38) A baseline risk for the onset of diabetes will be estimated using the QDiabetes algorithm. (23)

3a. Simulating adherence to treatment under usual care

The RUPEE study will evaluate the effect of adherence to medication on long term costs and health outcomes measured using quality adjusted life years (QALYS). The average rates of adherence in clinical trials can be higher than in actual practice (4) as seen in the UMPIRE clinical trial population which had an atypically high baseline adherence rate. Instead, adherence rates to medication (antihypertensives, statins and aspirin) under a usual care setting will be sourced from the 2011 HSE dataset. Participants in the 2011 HSE self-reported all the prescribed medications they had taken in the last 7 days. This was coded in the HSE dataset using the British National Formulary (BNF) classifications codes. Using this data, we are able to identify the medication patients were prescribed and identify whether or not they were taking the prescribed medication in the last week. This will reflect adherence to medication in a usual care population. The data will be used to estimate the probability of each person being adherent or not to medication. Individual characteristics will be used as predictors of adherence; the characteristics will be chosen by referring to studies which have assessed predictors of adherence in persons taking treatment for CVD. (39, 40) A generalized linear mixed regression model will be used to estimate the probability of adherence to medication for each individual. The probability of persistence with medication will not be assumed to be constant, and the model will include a probability of ceasing medication over time. The probability of medication cessation will be sourced from published literature on adherence.

3b. Estimate relative risks of adherence to medication

For peer review only - http://bmjopen.bmj.com/site/about/guidelines.xhtml

We will estimate the relative risks of adherence to medication, using a generalised linear mixed regression model which will be applied to the UMPIRE trial dataset (UK dataset). In the polypill scenario in the model, the probability of being adherent to medication will be further modified by the relative risks.

4. Adjust risk of events for treatment

We will source data on the treatment effects of statins, antihypertensives and aspirin from meta-analyses of intention-to-treat RCTs. Intention-to-treat analyses account for non-adherence in their findings, and therefore underestimate the impact of treatment on event risk. To overcome this, we will carry out sensitivity analyses to test the impact of adjusting for adherence within the trial. The risk of a CVD event will be adjusted by the relative risk of treatment with statins, antihypertensives and aspirin, based on the medication(s) the person is taking and whether or not they are adherent to medication.

5. Simulation of events

Individuals in the model can experience a CVD event at a rate governed by their calculated baseline risk (estimated by the QRISK2 or REACH algorithms) and adjusted for treatment effects if they have been simulated as adherent to treatment. CVD events will be categorised as a TIA, stroke, MI or angina. The relative incidence of each CVD event will be determined using published incidence data.(41) Similarly, the risk of new onset diabetes will be calculated using the QDiabetes algorithm. We will simulate the incidence of adverse events as a result of treatment: new onset diabetes and gastrointestinal bleeds. Data on the probability of an adverse event will be sourced from meta-analyses of randomised controlled trials for the relevant drugs. Mortality risk will be modelled as mortality from stroke and MI and other cause mortality. Data on other cause mortality will be estimated using national life tables for England and Wales. (42)

6. Assign cost and quality of life values

Costs and QALYs associated with each individual's simulated lifetime profile of CVD and related care will be estimated. Costs and quality adjusted life years (QALYs) will accrue for each person to reflect events, such as a stroke or new onset diabetes. Costs and utility values for health events will be sourced from published studies including the NICE guidelines for lipid modification and hypertension. (1,35,43) Costs of medication will be sourced from the NHS National Drug Tariff.(44)

For peer review only - http://bmjopen.bmj.com/site/about/guidelines.xhtml

7. Change in age, treatment, CVD status and type II diabetes status

The simulation model will run for each individual for lifetime duration (death or maximum age of 100 years) and patient characteristics will be updated after each event or every 10 years (depending on which event occurs first). A 10 year update is used as the QRISK2 algorithm returns a 10 year CVD risk.

For peer review only

For peer review only - http://bmjopen.bmj.com/site/about/guidelines.xhtml

Table 1) Input parameters

**Model inputs**	**Source**
**1. Individual dataset**	
Population dataset	Initial patient characteristics (see Figure 2) for cohort of patients drawn from a representative national sample: Health Survey for England (HSE) dataset 2011. The dataset will include patients who meet the entry criteria for the UMPIRE trial.
**2. Calculation of baseline risks**	
Risk calculators	Risk of first CVD event and onset of type 2 diabetes estimated for individuals using QRISK2 and QDiabetes.(23, 24)<br><br>*QRISK2: 10 year CVD risk (CVD outcomes defined as angina, MI, TIA and stroke)*<br>*QDiabetes: risk of acquiring type 2 diabetes over 10 year time period*<br><br>Risks for subsequent CVD events estimated for individuals using the REACH algorithm. (38)<br>*CVD outcomes defined as cardiovascular death (includes fatal stroke and MI), non-fatal MI, non-fatal stroke, and cardiovascular hospitalisation (includes hospitalisation for unstable angina and TIA)*
Risk of heart failure	Baseline risk per age using incidence rates in Cowie et al. (1998) (45)
Relative incidence of CVD events (TIA, stroke, angina, MI)	OXVASC cohort study, Rothwell *et al.* 2005. 91,106 individuals presenting with an acute vascular event in Oxfordshire, UK in 2002-5. (41)
**3. Adherence to medication**	
Probability of adherence to treatment with usual care	Estimates from HSE 2011 dataset on adherence to relevant drugs (statins, antihypertensives, aspirin)
Relative risk of adherence: polypill versus usual care	Estimate the probability of adherence to ≥2 antihypertensives, statin or anti-platelet for at least four days in the preceding week for polypill group versus usual care by applying a binomial regression to the UMPIRE dataset.
**4. Treatment effects of medication (antihypertensives, statin, anti-platelet)**	
Relative risk of CVD with treatment versus no treatment	For base case analysis, conventional meta-analysis of ITT RCT data will be used from –<br>▪ Cholesterol Treatment Trialists' Collaboration<br>▪ Blood Pressure Lowering Treatment Trialists' Collaboration<br>▪ Antithrombotic Trialists' Collaboration<br>▪ Law and Wald (46)<br>Sensitivity analysis: Test impact of adjusting for adherence within trials
**5. Other treatment outcomes (beneficial events and adverse events) and mortality rates**	
Adverse Events	
Incident type 2 diabetes	Relative risk of diabetes from statins/antihypertensives from meta-analyses of RCTs
GI bleeding	Relative risk of bleeding resulting from aspirin using estimates from meta-analyses of RCTs
Cough	Placebo-adjusted relative risk of cough resulting from ACE inhibitors using estimate from meta-analyses of RCTs

For peer review only

For peer review only - http://bmjopen.bmj.com/site/about/guidelines.xhtml

Reduction in heart failure	Relative risk reduction in heart failure from antihypertensives (33)
Mortality	
Stroke case fatality (60 day)<br>Age <75<br>Age > 75+	Estimate proportion of strokes that are fatal (with risks increasing with age). Estimate using the BHF Compendium of health statistics 2012, which has data from a record linkage study for England 2010.
MI case fatality (30 day)<br>Age 30-54<br>Age 55-64<br>Age 65-74<br>Age 75-84<br>Age 85+	Proportion of MI's that are fatal. Estimate using Oxford Record Linkage pill study. (47) National population based study, including all individuals admitted to hospital or who died suddenly from acute MI in 2010. Age was strongest predictive factor for 30-day case fatality.
Death from other causes	Estimated from national life tables (Office for National Statistics, England)(42)
**6. Costs (medication, monitoring costs, health events)**	
Drug costs (£ per year)	
Statins	National Health Service (NHS) Electronic Drug Tariff (44)
AHT drugs	
Aspirin	
Polypill	Assumed to be aggregate cost of each drug in the combined pill
Yearly monitoring costs while on medication	
Primary care nurse (£ per hour)<br>GP cost (£ per hour)<br>Lipid test (£)	Use NICE Quality Outcomes Framework to identify recommended management while on treatment (statins, antihypertensives, antiplatelet). A cost for stopping medication will also be applied (e.g. 2 GP visits, tests as recommended in NICE clinical guidelines 181) (1)
Liver transaminase test<br>Blood tests	Costs sourced from Personal Social Services Research Unit Costs and NICE clinical guidelines 181
Costs of health states and adverse events	
Stroke	Luengo-Fernandez *et al.* 2012 (48)
TIA	"
MI	NICE lipids guideline 181 (1)
Angina	"
PAD	"
Diabetes	"
GI bleeding	"
Cough (from ACE inhibitor use)	NICE Hypertension guidelines 127 (43)
**7. Health Related Quality of life**	
Stroke	Derived from Health Survey from England (HSE) dataset
TIA	
MI	
Angina	
PAD	
GI bleeding	
Diabetes	
Cough	

CVD: cardiovascular disease, TIA: transient ischaemic attack, MI: myocardial infarction; ITT: Intention to treat, RCT: randomised controlled trial, AHT: antihypertensives, UMPIRE:Use of a Multidrug Pill in

For peer review only - http://bmjopen.bmj.com/site/about/guidelines.xhtml

Reducing Cardiovascular Events, NICE: National Institute for Health and Care Excellence, GP:general practitioner

For peer review only

For peer review only - http://bmjopen.bmj.com/site/about/guidelines.xhtml

Analysis

The simulation model will run for a sufficient number of iterations to provide stable results. Uncertainty in the model parameters will be examined using a probabilistic sensitivity analysis (PSA) which will reflect uncertainty over the values of the model inputs. Non-parametric bootstrapping of HSE data will be carried out to examine the uncertainty related to the sampling. For each PSA iteration, one non-parametric bootstrap sample will be drawn from the HSE dataset (by random sample with replacement of individuals in the dataset). An incremental analysis will be conducted and incremental cost-effectiveness ratios (ICERs) and net benefit statistics will be estimated. We will also carry out a number of sensitivity analyses to test the impact of varying uncertain parameters in the model. This will include an analysis testing the impact of varying the polypill cost.

Validation

The model will be internally and externally validated. A checklist produced by the RUPEE steering group based on current published guidelines for checking models will be used, to ensure the programmed model behaves as expected according to the theoretical model. (21, 49) The checklist includes tips for model developers, for example on the use of sensitivity analyses to test that the model is operating correctly, and re-programming complicated sections of code in another language. The model will also be reviewed and tested by an experienced modeller. The model results will be compared with real-world observations or the results of other models.

Dissemination of results

The findings of the economic evaluation will be presented to scientific and health care policy audiences in open access journals and at national and international conferences. We will also present findings to NHS policy makers and pharmaceutical companies.

DISCUSSION

Medication adherence is important for disease management, and benefits of increased adherence to preventative medication for CVD include improved clinical outcomes. (5) The UMPIRE clinical trial was conducted to evaluate the effect of a polypill strategy compared to usual care on adherence. It showed that the polypill strategy significantly augmented adherence and this was reflected by improvements in SBP and LDL-C. (8) Whether or not this impact remains in the long term cannot be determined from the trial data alone. The RUPEE (NHS) study is being conducted to evaluate the long term impact of a polypill strategy; in particular, the analysis will evaluate the long term impact of increased adherence on outcomes. An economic model is being developed to estimate the long term costs and QALYs associated with implementing a polypill strategy in the NHS compared to usual

For peer review only - http://bmjopen.bmj.com/site/about/guidelines.xhtml

For peer review only

care. This analysis will represent the first comprehensive cost effectiveness analysis using directly applicable clinical trial data.

This paper outlines the process behind the design of the economic model. We carried out a review of published CVD models to identify a modelling approach that would suit the health care decision: use of a polypill versus usual care in a population with or at high risk of CVD. We identified an individual simulation model as the most appropriate approach as it allows the heterogeneity in the population to be adequately reflected. The model will use validated disease risk algorithms to estimate the probability of an individual experiencing a CVD event or the onset of diabetes. Individuals can also experience an increased risk of an adverse event (diabetes, cough and gastrointestinal bleeding) from treatment. The risk of a CVD event will be reduced if the individual is adherent to treatment. We will simulate adherence to treatment using data from the HSE 2011 dataset. The probability of adherence in the polypill scenario will be further modified by the relative risks of adherence to medication which will be sourced from the UMPIRE trial data for the English population. Costs and QALYs will be estimated for each individual and aggregated across the sample population (based on the HSE 2011 dataset).

The RUPEE (NHS) model will have a number of advantages over existing models constructed to evaluate a CVD polypill. (50-52) One advantage is the use of an individual simulation model which will allow us to capture the heterogeneity in the variation in CVD risk in the UK population unlike other models which use Markov type transition state models. Another is that we will extrapolate data on adherence to medication from a nationally representative population dataset (Health Survey for England) which will allow us to simulate adherence per individual rather than assuming a constant adherence across our population. We will also allow for adverse events from treatment and treatment cessation, therefore more accurately reflecting clinical practice.

It would be preferable to use per protocol treatment effectiveness data in our analysis as intention-to-treat data already accounts for adherence (people switching and ceasing medication during the trial period). However, per protocol data is difficult to obtain for all drugs, therefore we will use the ITT treatment effect data and carry out sensitivity analyses to test the impact.

The introduction of a CVD preventive polypill strategy will simplify pill taking for patients potentially leading to greater adherence and better health outcomes. This analysis will provide information on the cost-effectiveness of the polypill in a NHS setting.

For peer review only - http://bmjopen.bmj.com/site/about/guidelines.xhtml

Contributors: CC carried out the literature review and drafted the manuscript. JL, ST, HMD, HW and NP, AR and SJ contributed to the development of the protocol. JL provided input on the heath economics model. HMD provided statistical advice and ST, HW and NP contributed clinical advice. AR and SJ peer reviewed draft manuscripts and contributed to the final version of the protocol. All authors approved the final version of the manuscript submitted for publication.

Funding: This paper presents independent research funded by the National Institute for Health Research (NIHR) under its Research for Patient Benefit (RfPB) Programme (Grant Reference Number PB-PG-1112-29080). The views expressed are those of the author(s) and not necessarily those of the NHS, the NIHR or the Department of Health.

Competing interests: ST, NP and HW acknowledge support from the NIHR Biomedical Research Centre at Imperial College Healthcare NHS Trust and Imperial College London. SJ and AR are employed by The George Institute for Global Health who own the IP for the polypill in Australia.

Ethics approval: No ethics approval was required

For peer review only

References

(1) National Institute for Health and Care Excellence (NICE*)*,. Lipid modification: cardiovascular risk assessment and the modification of blood lipids for the primary and secondary prevention of cardiovascular disease. 2014;Clinical Guideline 181.

(2) JBS3 Board. Joint British Societies' consensus recommendations for the prevention of cardiovascular disease (JBS3). *Heart* 2014 Apr;100 Suppl 2:ii1-ii67.

(3) Yusuf S, Islam S, Chow CK, et al. Use of secondary prevention drugs for cardiovascular disease in the community in high-income, middle-income, and low-income countries (the PURE Study): a prospective epidemiological survey. *The Lancet* 2011;378(9798):1231-1243.

(4) Osterberg L, Blaschke T. Adherence to medication. *N Engl J Med* 2005;353(5):487-497.

(5) Simpson RJ, Mendys P. The effects of adherence and persistence on clinical outcomes in patients treated with statins: a systematic review. *Journal of clinical lipidology* 2010;4(6):462-471.

(6) Connor J, Rafter N, Rodgers A. Do fixed-dose combination pills or unit-of-use packaging improve adherence? A systematic review. *Bull World Health Organ* 2004;82(12):935-939.

(7) Elley CR, Gupta AK, Webster R, et al. The efficacy and tolerability of 'polypills': meta-analysis of randomised controlled trials. *PLoS One* 2012;7(12):e52145. doi: 10.1371/journal.pone.0052145

(8) Thom S, Field J, Poulter N, et al. Use of a Multidrug Pill In Reducing cardiovascular Events (UMPIRE): rationale and design of a randomised controlled trial of a cardiovascular preventive polypill-based strategy in India and Europe. *Eur.J.Prev.Cardiol*. 2014; 21(2) 252-261

(9) Thom S, Poulter N, Field J, et al. Effects of a fixed-dose combination strategy on adherence and risk factors in patients with or at high risk of CVD: the umpire randomized clinical trial. *JAMA* 2013;310(9):918-929.

(10) Caro JJ, Briggs AH, Siebert U, et al. ISPOR-SMDM Modeling Good Research Practices Task Force. Modeling good research practices--overview: a report of the ISPOR-SMDM Modeling Good Research Practices Task Force-1. *Med Decis Making* 2012 Sep-Oct;32(5):667-677.

(11) Roberts M, Russell LB, Paltiel AD, et al. Conceptualizing a model: a report of the ISPOR-SMDM Modeling Good Research Practices Task Force-2. *Med Decis Making* 2012 Sep-Oct;32(5):678-689.

(12) National Institute for Health and Care Excellence (NICE),. Guide to the methods of technology appraisal 2013.

(13) Barton P, Andronis L, Briggs A,et al. Effectiveness and cost effectiveness of cardiovascular disease prevention in whole populations: modelling study. *BMJ* 2011 Jul 28;343:d4044.

(14) Weinstein MC, Coxson PG, Williams LW,et al. Forecasting coronary heart disease incidence, mortality, and cost: the Coronary Heart Disease Policy Model. *Am J Public Health* 1987 Nov;77(11):1417-1426.

For peer review only - http://bmjopen.bmj.com/site/about/guidelines.xhtml

(15) Cooper K, Davies R, Roderick P, et al. The development of a simulation model of the treatment of coronary heart disease. *Health Care Manag Sci* 2002;5(4):259-267.

(16) Whitfield MD, Gillett M, Holmes M, et al. Predicting the impact of population level risk reduction in cardio-vascular disease and stroke on acute hospital admission rates over a 5 year period—a pilot study. *Public Health* 2006;120(12):1140-1148.

(17) Unal B, Capewell S, Critchley JA. Coronary heart disease policy models: a systematic review. *BMC Public Health* 2006 Aug 18;6:213.

(18) Capewell S, Allender S, Critchley J, et al. Modelling the UK burden of cardiovascular disease to 2020: a research report for the Cardio & Vascular Coalition and the British Heart Foundation. *British Heart Foundation* 2008(Available at http://www.healthimpact.org.uk/content/resources/z154_modelling_the_burden_of_cv_disease.pdf).

(19) Hotchkiss JW, Davies C, Gray L, et al. Trends in adult cardiovascular disease risk factors and their socio-economic patterning in the Scottish population 1995-2008: cross-sectional surveys. *BMJ Open* 2011 Aug 9;1(1):e000176-2011-000176.

(20) Ward S, Jones ML, Pandor A, et al. A systematic review and economic evaluation of statins for the prevention of coronary events. 2007. Health Technology Assessment 2007; 11(14):1-160, iii-iv.

(21) Davis S, Stevenson M, Tappenden P, et al. NICE DSU Technical Support Document 15: Cost-effectiveness modelling using patient level simulation. 2014.

(22) Hippisley-Cox J, Coupland C, Vinogradova Y, et al. Predicting cardiovascular risk in England and Wales: prospective derivation and validation of QRISK2. *BMJ* 2008 Jun 28;336(7659):1475-1482.

(23) Hippisley-Cox J, Coupland C, Robson J, et al. Predicting risk of type 2 diabetes in England and Wales: prospective derivation and validation of QDScore. *BMJ* 2009 Mar 17;338:b880.

(24) Hippisley-Cox J, Coupland C. Unintended effects of statins in men and women in England and Wales: population based cohort study using the QResearch database. *BMJ*2010;340.

(25) Macedo AF, Taylor FC, Casas JP, et al. Unintended effects of statins from observational studies in the general population: systematic review and meta-analysis. *BMC Med* 2014 Mar 22;12:51-7015-12-51.

(26) Elliott WJ, Meyer PM. Incident diabetes in clinical trials of antihypertensive drugs: a network meta-analysis. *The Lancet* 2007;369(9557):201-207.

(27) Gupta AK, Dahlof B, Dobson J, et al. Determinants of new-onset diabetes among 19,257 hypertensive patients randomized in the Anglo-Scandinavian Cardiac Outcomes Trial--Blood Pressure Lowering Arm and the relative influence of antihypertensive medication. *Diabetes Care* 2008 May;31(5):982-988.

(28) Antithrombotic Trialists' Collaboration. Collaborative meta-analysis of randomised trials of antiplatelet therapy for prevention of death, myocardial infarction, and stroke in high risk patients. *BMJ* 2002 Jan 12;324(7329):71-86.

For peer review only - http://bmjopen.bmj.com/site/about/guidelines.xhtml

For peer review only

(29) Baigent C, Blackwell L, Collins R, et al. Antithrombotic Trialists'(ATT) Collaboration. Aspirin in the primary and secondary prevention of vascular disease: collaborative meta-analysis of individual participant data from randomised trials. *The Lancet* 2009;373(9678):1849-1860.

(30) Peters R, Beckett N, Burch L, et al. The effect of treatment based on a diuretic (indapamide) ± ACE inhibitor (perindopril) on fractures in the hypertension in the very elderly trial (HYVET). *Age Ageing* 2010;39(5):609–16.

(31) SPRINT. A Randomized Trial of Intensive versus Standard Blood-Pressure Control. *N Engl J Med* 2015; 373:2103-2116

(32) Finegold J A, Manisty CH, Goldacre B, et al. What proportion of symptomatic side effects in patients taking statins are genuinely caused by the drug? Systematic review of randomized placebo-controlled trials to aid individual patient choice. *Eur J Prev Cardiol*. 2014; 21: 464

(33) Ettehad D, Emdin CA, Kiran A, et al. Blood pressure lowering for prevention of cardiovascular disease and death: A systematic review and meta-analysis. *The Lancet.* 2015;387(10022):957–67

(34) Rothwell PM, Fowkes FGR, Belch JFF, et al. Effect of daily aspirin on long-term risk of death due to cancer: analysis of individual patient data from randomised trials. *The Lancet.* 2011; 377(9759):31-

(35) National Institute for Health and Care Excellence (NICE),. Lipid Modification Cardiovascular risk assessment and the modification of blood lipids for the primary and secondary prevention of cardiovascular disease. *NICE Clinical Guidelines* 67 2008 (Modified 2010).

(36) D'Agostino RB, Vasan RS, Pencina MJ, et al. General cardiovascular risk profile for use in primary care the Framingham Heart Study. *Circulation* 2008;117(6):743-753.

(37) Collins GS, Altman DG. An independent and external validation of QRISK2 cardiovascular disease risk score: a prospective open cohort study. *BMJ* 2010;340.

(38) Wilson PW, D'Agostino R, Bhatt DL, et al. An international model to predict recurrent cardiovascular disease. *Am J Med* 2012;125(7):695-703. e1.

(39) Mann DM, Woodward M, Muntner P, Falzon L, Kronish I. Predictors of Nonadherence to Statins: A Systematic Review and Meta-Analysis. *Ann Pharmacother. 2010;* 44:1410-21

(40) Baker A, Chen LC, Elliott R. Factors Associated with Patients' Adherence to Antihypertensive Drug Classes in the UK. *PHARMACOEPIDEMIOLOGY AND DRUG SAFETY* 2014; 23: 320-320.

(41) Rothwell P, Coull A, Silver L, et al. Population-based study of event-rate, incidence, case fatality, and mortality for all acute vascular events in all arterial territories (Oxford Vascular Study). *The Lancet* 2005;366(9499):1773-1783.

(42) Office for National Statistics. Available at: http://www.ons.gov.uk/ons/taxonomy/index.html?nscl=Life+Tables.

For peer review only

For peer review only - http://bmjopen.bmj.com/site/about/guidelines.xhtml

(43) National Institute for Health and Clinical Excellence,. Hypertension: clinical management of primary hypertension in adults. 2011;*Clinical Guideline 127*.

(44) National Health Services (NHS) Business Services Authority Drug Tariff. Available at: http://www.nhsbsa.nhs.uk/PrescriptionServices/4940.aspx.

(45) Cowie MR WDCATSP-WPSVaSG. Incidence and aetiology of heart failure. A population based study. European Heart Journal. 1999; 20: 421-428

(46) Law M, Morris J, Wald N. Use of blood pressure lowering drugs in the prevention of cardiovascular disease: meta-analysis of 147 randomised trials in the context of expectations from prospective epidemiological studies. *BMJ* 2009;338. 37

(47) Smolina K, Wright FL, Rayner M, et al. Determinants of the decline in mortality from acute myocardial infarction in England between 2002 and 2010: linked national database study. *BMJ* 2012;344. 38

(48) Luengo-Fernandez R, Gray AM, Rothwell PM. A Population-Based Study of Hospital Care Costs During 5 Years After Transient Ischemic Attack and Stroke. *Stroke* 2012;43(12):3343-3351. 39

(49) Philips Z, Bojke L, Sculpher M, et al. Good practice guidelines for decision-analytic modelling in health technology assessment. *Pharmacoeconomics* 2006;24(4):355-371.

(50) Becerra V, Gracia A, Desai K, et al. Cost-effectiveness and public health benefit of secondary cardiovascular disease prevention from improved adherence using a polypill in the UK. *BMJ Open* 2015 May 9;5(5):e007111-2014-007111.

(51) Newman J, Grobman WA, Greenland P. Combination Polypharmacy for Cardiovascular Disease Prevention in Men: A Decision Analysis and Cost-Effectiveness Model. *Preventive cardiology* 2008;11(1):36-41.

(52) van Gils PF, Over EAB, Hamberg-van Reenen HH, et al. The polypill in the primary prevention of cardiovascular disease: cost-effectiveness in the Dutch population. *BMJ Open* 2011;1:e000363 doi:101136/bmjopen-2011-000363

For peer review only

Figure 1 Flowchart of Literature Review of cardiovascular disease models

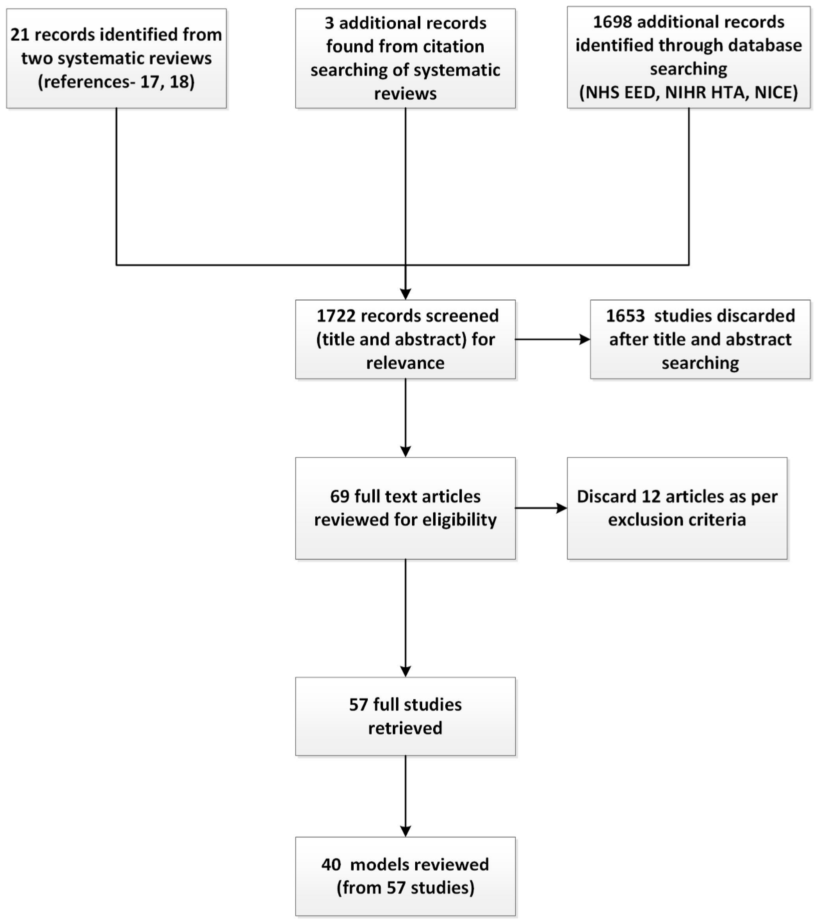

NHS EED: National Health Service Economic Evaluation Database,
NIHR HTA: National Institute for Health Research Health Technology Assessment,
NICE: National Institute for Health and Care Excellence

Figure 1 Flowchart of literature review search for cardiovascular disease models

190x258mm (300 x 300 DPI)

Figure 2 RUPEE (NHS) simulation model flowchart

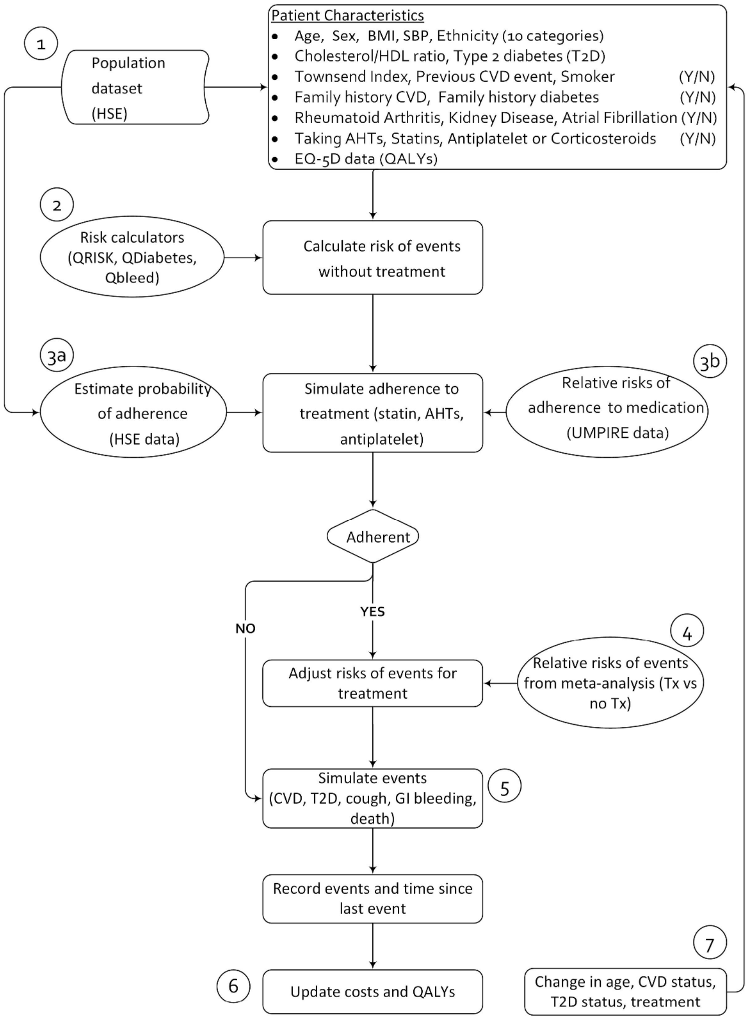

HSE: Health Survey for England, T2D: Type 2 Diabetes, CVD: cardiovascular disease, AHTs: antihypertensives, tx: treatment, GI: gastrointestinal, QALYs: quality adjusted life years

Figure 2 RUPEE (NHS) Simulation model flowchart

234x326mm (300 x 300 DPI)

Supplementary Appendix Figure 1 Illustration and details of NICE Clinical Guidelines 181 Markov Model

Markov model illustration CG 181

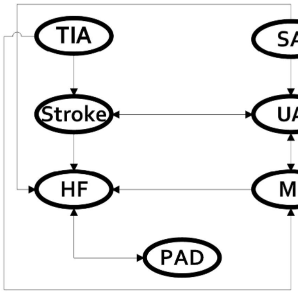

Markov Model also includes:
- Risk of death from CVD and all cause mortality
- Each health state also has a post health state to compensate for memoryless limitations of Markov modelling

Population
- England and Wales
- Adults without CVD
- Adults with established CVD
- Adults with type 2 diabetes & without CVD
- Male & Female (40 +) CVD, diabetes baseline risk set at 5-30% (QRISK2 & UKPDS)

Baseline Risk
Primary Prevention:
- Risk of first CVD event estimated using annual incidence CVD event/total incidence of those events
- Risk of first event updated annually by fixed amount each year (represent increase with age) – 0.03% for males and 0.008% for females

Secondary Prevention:
- Absolute incidence converted into proportion of events- SA, UA, MI, TIA, Stroke, HF, PAD

Medication
- Low, medium and high intensity statins evaluated

Treatment effectiveness
- Treatment effect applied as relative reduction in CVD events and mortality
- Relative risks were applied for each statin intensity group based on clinical review carried out for guideline
- Same treatment effect assumed for primary and secondary prevention
- Equal relative effectiveness assumed for patients of all ages regardless of baseline risk
- Clinical effectiveness continues throughout life

Adverse events
Early onset diabetes:
- Secondary prevention- 10% overall, 0.9% early onset
- Primary prevention- 5% overall, 0.45% early onset
- Myalgia
- liver dysfunction

Adverse events assumptions
- Adverse events (except diabetes) are temporary and reversible
- Switch to another statin if liver dysfunction or myalgia
- Rhabdomyolysis not included
- Model did not include costs for myalgia and liver dysfunction events

TIA: transient ischemic stroke, SA: stable angina, UA: unstable angina, HF: heart failure, MI: myocardial infarction, PAD: peripheral arterial disease, CVD: cardiovascular disease, QRISK2: Algorithm to estimate cardiovascular disease, UKPDS: UK Prospective Diabetes Study Risk Engine,

SupplementaryAppendix Figure 1 Illustration and details of Markov model developed for NICE clinical guidelines 181

209x293mm (300 x 300 DPI)

Supplementary appendix

Section 1- Literature review

The purpose of the literature review was to identify the appropriate analytical framework to represent the decision problem. The literature review also aimed to inform the underlying model structure: disease and care pathway.

A general search of the literature identified a known review of coronary heart disease policy models by Unal et al. (2006).(1) This review was updated and expanded in 2008 by Capewell et al. to include stroke models. (2) A search carried out in Medline found no further systematic reviews of coronary heart disease or cardiovascular disease models published since 2008. The review by Capewell et al. (2008) identified seven notable CHD models (six of these had been identified in the previous review by Unal et al), nine stroke models and several models that were currently in development at the time of publication. The notable models and models in development were reviewed. Additionally, citation searching of both reviews was carried out to potentially identify any further models published since 2008.

Development of search strategy

The purpose of the review was not to identify every single model for cardiovascular disease but rather to identify potential model structures that could be adapted or used to help construct the RUPEE NHS model. Initially, it was planned that an updated search using the search strategy devised by Unal et al. (2006) and updated by Capewell et al. (2008) would be used. However, the purpose of both reviews had been to identify notable policy models at the population level. It was felt that redoing this review could potentially fail to return other models which could be used such as those developed for NICE guidelines. The choice of databases was discussed with a systematic reviewer based at HERG. The search strategy was carried out using the following databases:

- NHS economic evaluation Database (NHS EED): this database contains economic evaluations of healthcare interventions and is produced by the NIHR Centre for Reviews and Dissemination (CRD) at the University of York, UK.
- National Institute for Health Research (NIHR) Health Technology Assessment (HTA) monograph series: This series publishes research about the effectiveness, costs and broader impact of healthcare treatments and tests (within a UK National Health Service (NHS) setting).
- National Institute for health and care excellence (NICE) website: this database publishes evidence based guidance on preventative, diagnostic and treatment interventions for disease and ill health.

For peer review only - http://bmjopen.bmj.com/site/about/guidelines.xhtml

NHS EED was identified as an appropriate database as this database reviews and produces critical commentaries economic evaluations of 'key' relevance to the UK NHS. The critical commentaries provide a summary of the overall reliability and generalisability of the study. The NICE HTA monograph series publishes research including cost-effectiveness analyses of healthcare treatment and tests; the series was searched to identify published HTA's which have developed or used a cardiovascular disease model. The NICE guidelines website was searched to identify guidelines related to cardiovascular disease (for example guidelines for lipid modification).

The search terms used in the search included 'cardiovascular disease', 'coronary heart disease', 'stroke', 'myocardial infarction', 'angina' and 'peripheral artery disease'. Appendix 1 contains further details of the searches carried out in each database.

Exclusion criteria

Studies were excluded if they did not discuss the development or review of an economic model; if no disease states for cardiovascular disease were included in the model; if the focus of the study was a diagnostic test or surgical intervention where the focus of the evaluation was a short term follow up, (<10 years). Studies were not excluded on the basis of intervention (treatment or lifestyle intervention) or on the basis of date published or language.

Data extraction form

The use of a standard checklist such as the Drummond economic evaluation checklist was considered to review each study but was found to be unsuitable for reviewing the models as the design of the checklist leads the reviewer to evaluate the cost-effectiveness analysis inputs and outcomes with only two questions referring to the model structure: regarding the choice and details of the model. (3)

Therefore, a data extraction form was designed to extract data that was required to meet the purpose of the review. An initial data extraction form was developed which extracted data on the following items:

- Paper (Author, Year)
- Purpose of the Model
- Setting and Population
- Interventions
- Type of model (Simulation, Markov Model, other)
- Brief description of Model
- Cardiovascular disease risk algorithms

For peer review only - http://bmjopen.bmj.com/site/about/guidelines.xhtml

- Risk factors included to calculate cardiovascular disease risk
- Disease stages (Health states) included in model
- Source of data inputs used in model (Population data, mortality rate, treatment uptake and effectiveness, other)
- Probabilistic Distributions and Parameters

The form was refined further to only extract data which was relevant for this review. As the purpose of the review was to inform the model structure and design the extraction of data inputs and probabilistic distributions and parameters were removed from the data extraction form. The initial data extraction form also extracted data on the quality of each model. An assessment of quality criteria for models has been suggested in guidelines from the International Society for Pharmaeconomics and Outcomes Research (ISPOR). (4,5) The systematic review by Unal et al. used the guidelines suggested by ISPOR to create a grading system for model papers based on the sensitivity, validity and transparency of a model. As the purpose of the review is not to evaluate inputs, the form was further refined and information on sensitivity analyses were not extracted. However, the data extraction forms did extract information on whether the model had been validated (including details of validation). The refined data extraction form also included a section on whether the model had been adapted for further studies. An example of a completed data extraction form can be found in Section 2.

Categorisation of Models

Each model was categorised (modelling approach) based on the taxonomy of model structures as developed by Brennan et al. (6)

Findings

The majority of models identified for review used a state transition approach (13 models) with five models adopting a hybrid state transition, in all cases a hybrid Markov-simulation model. (7-24) Only one decision tree model was identified, whereas 10 individual simulation model were identified. (25-35) Another popular approach was to use a systems dynamics modelling approach (5 models).(36-40) Other modelling approaches identified included an age period cohort (APC) approach (1 model); a tabular cell based model used by the World Health Organisation to estimate the global burden of disease; two life table approaches; a mathematical stroke epidemiological model and the Archimedes model which uses a method based on Fourier expansions using standard mathematical techniques to simulate individuals (proprietary model). (41-46)

The identified models categorised according to modelling approach can be found in Table 1.

For peer review only - http://bmjopen.bmj.com/site/about/guidelines.xhtml

The purpose of the review was to identify the best modelling approach for the RUPEE study. To this end, we reviewed the models to identify advantages and disadvantages of each approach. Details of the advantages and disadvantages of the two main modelling approaches used (Markov models and Simulation models) can be found in the paper associated with this supplementary appendix. Table 2 presents a summary of six models which used a different modelling approach (decision tree, state transition model, simulation model, systems dynamic and hybrid model).

Additionally, schematic illustrations of several models were used to aid discussions about the different types of modelling approaches with clinical experts. Figure 1 in Section 2 is an example of the schematic illustration for the model developed for the NICE clinical guidelines 181 which evaluated statin treatment in primary and secondary care. (47)

For peer review only

For peer review only - http://bmjopen.bmj.com/site/about/guidelines.xhtml

Table 1- Models identified in Literature Review Search

Life Table/Cell base/Tabular model	Decision Tree	State Transition	Hybrid model	Simulation	Systems Dynamic/ Compartmental model
WHO Global Burden of Disease (42)	Whitfield et al. (UK) (25)	Grover et al. CVD Life Expectancy model (Canada)- Markov Model (7)	Rotterdam Ischemic disease and stroke (RISC) model<br>Markov model structure with individual simulation (20)	Southampton CHD Policy Analysis Model 'Treatment' – individual simulation (26)	Weinstein et al. CHD heart disease policy model (USA) (36)
Schau et al. Stroke Model (Denmark)(45)		Stroke Treatment Economic Model (STEM)- USA (8)	Duke Stroke Policy and Prevention Model USA (SPPM)<br>Semi-Markov/simulation model (21)	CHD Policy Analysis Model 'Prevention'- individual simulation (27)	IMPACT model (including adaptations of model) (37)
Tobias et al. APC Model (41)		RIVM Chronic Disease- Markov Model (9)	A Dynamic modelling tool for generic health impact assessments (Dynamo-HIA)<br>Markov/partial simulation model (22)	Prevent – Macro simulation model using aggregated data (policy tool) (28)	Sundberg et al.- Compartmental model (38)
Struijs et al. Dynamic multi-state life table (43)		Ward et al . (ScHAAR statins model) and adaptations- Markov Model (10) (47)	Korean Individual Microsimulation Model for Cardiovascular Health Interventions<br>Hybrid Markov/ individual simulation model (23)	Foresight Obesity Model UK – stochastic cohort simulation approach (29)	Model of Resource Utilization, Costs and Outcomes for Stroke, (MORUCOS, Australia)- Compartmental model (39)
Archimedes (USA) (46)		Smith-Spangler et al- Markov Model (11)	Soresen et al. Simulation model<br>Markov model/individual simulation (24)	POHEM- Canada, Microsimulation (30)	PopMod: a longitudinal population model with two interacting disease

For peer review only

For peer review only - http://bmjopen.bmj.com/site/about/guidelines.xhtml

					states- Compartmental model (40)
Malik et al. Life Table (44)		Newman et al. Combination polypharmacy, Markov model 12)		EUROASPIRE III health economics project- Individual simulation (31)	
		Grosso et al, Markov Model 13)		OECD and WHO microsimulation chronic disease prevention simulation model- microsimulation (32)	
		Gillespie et al. SPHERE Markov Model (14)		Ara et al. Obesity model- Cohort simulation (33)	
		Wisloff et al. NorCaD Markov Model (15)		Department of Health Vascular Checks Model- Simulation (34)	
		Nash et al. Markov Model (16)		Green et al. Chronic Disease Policy Model- Discrete Event Simulation (35)	
		Lovibond et al. Markov Model (17)			
		Greving et al. Markov Model (18)			
		NICE Clinical Guidelines CG127 (19)			

WHO- World Health Organisation, APC- Age Period Cohort, CHD- Coronary heart disease, OECD- Organisation for Economic Co-operation and Development, ScHAAR- School of Health and Related Research, NICE- National Institute for Health and Care Excellence

For peer review only

For peer review only - http://bmjopen.bmj.com/site/about/guidelines.xhtml

Table 2- Summary of CVD models

Model name (Author)	Model Type	Risk factors	Health States/Events	Transparency & Validation	Limitations
Stroke Model (Whitfield et al.) (25)	Decision Tree	BMI, Type II diabetes, smoking, total and HDL cholesterol, SBP	Acute episode included: Acute CVD, Elective CVD, Heart Failure, Renal replacement procedures, Stroke, Diabetes (hypoglycaemia)	Internal validation: predicted number of CVD related admissions based on risk factor data compared to actual data (from five UK primary care trusts)- found results to be accurate<br><br>No external validation conducted	The model uses an aggregate approach despite having individual data<br><br>Short time frame also used, suitable for decision tree but potentially if a longer time frame was used this would not be a suitable model
NICE lipid modification guidelines economic model (CG181) (47)	Markov Model	Not explicitly stated	Death from cardiovascular cause and non CVD death, stable angina, unstable angina, myocardial infarction, transient ischaemic attack, heart failure, peripheral artery disease and post event states for each non-fatal event	Yes, the model structure, assumptions and inputs are clearly reported<br><br>Validation has not been stated, this is an update of a previously widely used model (ScHAAR statins model NICE TA94) (10)	The model is limited by the Markovian assumption of memoryless though it does have tunnel (post event) states. The cohort can experience each event only once<br><br>The model structure is not suitable to simulate a heterogeneous population
RISC state transition hybrid model (20)	Hybrid Markov Model	sex, age, smoking status, SBP & DBP, BMI, waist to hip ratio, ankle-brachial index, levels of plasma glucose, total cholesterol,	Well, Stroke, CHD, CHD & Stroke, Other Death, CVD death	Internal validation: cumulative incidences simulated by RISC model compared to Rotterdam study incidences- similar.	Allow for individual heterogeneity to be modelled, but limited by Markovian state transition model (progression

For peer review only

		HLD, creatinine, family history CVD, hypertension, taking antihypertensives or BP over 160/90, presence diabetes II, intermittent claudication, angina, AF, TIA or prevalent CVD		External validity tested- used NORFOLK EPIC dataset and simulation incidences using model- incidences similar	between states and handling of time). Could potentially be slow computationally to run (uses six transition probabilities equations per individual) if more health states or risk factors are required
IMPACT (Capewell et al) (37)	Compartmental/ systems dynamics model	Cigarette smoking, total cholesterol, systolic blood pressure, BMI, diabetes, physical activity and fruit and vegetable consumption	Deaths prevented or postponed from reductions in risk were the main CHD outcome<br><br>Nine patient groups were evaluated:<br><br>Patients treated in hospital for acute myocardial infarction (MI)<br><br>Patients admitted to hospital with unstable angina<br><br>Community dwelling patients who have survived a MI >1yr<br><br>Patients who had undergone a previous	A technical appendix was provided a recent paper which used the IMPACT model and this provided detailed information on the equations used to estimate deaths prevented or postponed from a treatment intervention or a reduction in CVD risk factors and provided all data sources that were used in the modelling	Cost and QALYs were not Considered<br><br>The model did not look at th reduction in CVD events, it w limited to avoided mortality from CHD<br><br>A recent expansion of the m (IMPACT 2) is available, how though online this model is a black box and a technical appendix was not available<br><br>IMPACT2 is a DES model

For peer review only - http://bmjopen.bmj.com/site/about/guidelines.xhtml

			revascularisation procedure<br><br>Community dwelling patients with coronary artery disease<br><br>Patients admitted to hospital with heart failure<br><br>Community dwelling patients with heart failure<br><br>Hypercholesterolaemic patients without CHD<br><br>Hypertensive patients without CHD		
CHD Policy Analysis Model – Prevention component (Babad et al). (27)	Discrete Event Simulation	Age, sex, SBP, total cholesterol and smoking	Onset of stable angina, unstable angina, myocardial infarction, sudden cardiac death, stroke death, other cardiovascular disease, cancer death and death from other or unknown cause (potential to include HDl cholesterol)	The model structure could be replicated – however no data inputs are given regarding treatment effectiveness	Use of Framingham study to estimate baseline risk- recent studies have shown that QRISK is more suited to a UK population<br><br>Computational requirements: Model was run in special software (POST, DELPHI framework). This type of model would be computationally intensive to run in widely available

For peer review only - http://bmjopen.bmj.com/site/about/guidelines.xhtml

					packages such as Microsoft Excel
Department of Health Vascular checks economic model (34)	Individual simulation	Age, gender, townsend score, BMI, SBP, Smoking status, Total cholesterol/HDL ratio, record family history of CHD	Not explicitly stated. Costs and health benefits applied in the model were based on published NICE guidance (PH1002, CG43, CG34 and TA94)	The model inputs and data inputs are clear	Cost and QALYs relating to interventions were not directly estimated: rather they were sourced from existing guidance and linked to the simulation outputs<br><br>Requirement for a suitable large dataset to simulate can be expensive. The Department of Health used the proprietary GP database QRESEARCH (approximate cost of dataset £15-20,000).

BMI-body mass index, HDL- high-density lipoprotein, SBP- systolic blood pressure, DBP- diastolic blood pressure, CVD-cardiovascular disease, CHD-coronary heart disease, BP- blood pressure, AF- atrial fibrillation, TIA- transient ischemic attack, NICE-National Institute for Health and Care Excellence, DES-discrete event simulation, MI-myocardial infarction, ScHAAR-School of Health and Related Research, RISC- Rotterdam Ischemic disease and stroke model

For peer review only - http://bmjopen.bmj.com/site/about/guidelines.xhtml

Section 2- Example of completed data extraction form

Model name: Southampton Disease Model (CHD Policy Analysis Model) 'Treatment Model'

Paper (Author, Year): The development of a simulation model of the treatment of coronary heart disease (Keith Cooper and Ruth Davies, 2002)

Journal: Health Care Management Science 5, 259-267

Model Details

Model Structure: Discrete Event Simulation

Model software: Patient orientated simulation technique (POST) software with a Delphi interface.

Study Population & Setting: Individuals with stable angina, unstable angina or myocardial infarction (till age 85 or death).

Purpose of Model: The model is used to evaluated revascularisation at a hospital level rather than population based. Looks at progress of patients after a coronary event.

Patient characteristics: Given attributes of age, gender, vessel disease, time before cardiac death and time to age 85.

Model Description: New patients enter the model with SA, UA or MI (proportion randomly determined using incidence rate of disease). The following assumptions are employed:

- Risk of non-cardiac death
- Risk SA or UA leads to risk of MI
- SA leads to risk of UA
- Sampled time to event (MI, death, UA) depend on age and vessel disease
- Risks of UA, MI, & death increase with age, severe vessel disease and with a history of previous myocardial infarctions.
- Risks are independent of each other and are multiplied by baseline risks to change the projections of MI and death.
- Time updated Gompertz distribution (hazard function) used to estimate time to event (includes relative risks from vessel disease, prior history and interventions)

For peer review only

For peer review only - http://bmjopen.bmj.com/site/about/guidelines.xhtml

Progression to health events: Stable angina- Start in GP state receiving treatment (medical), assuming some have symptoms controlled and some not. Some are transferred to outpatient's investigations (now or in x years). After outpatients, some join a waiting list for an angiogram (queue) and those who do not go to a medical treatment stage. Vessel disease extent will determine next step after angiogram (can change this rule/input in simulation). Patients can bypass graft, angioplasty. Incidence data from Health Survey for England and GP Morbidity data.

Validation of model: validated cardiac deaths against mortality data from Office for National Statistics, based on death certificates. Model did underestimate deaths in females. Authors surmised this was due to poor reporting of causes of death on certificates.

Limitations Study (2002) does not mention the application of costs or QALYs and it looks at CHD events only. The authors noted that the model will be developed further to link the outputs to costs and to include secondary prevention such as aspirin or anti-cholesterol agents and to link the treatment with the prevention model (Prevent model developed by Babad et al.)

Summary- Discrete event simulation model for progress of patients after a coronary event. Individuals have angina and can progress to unstable angina or myocardial infarction. Changes in risks in one part can affect other parts of model. This model did allow for resource constraints such as availability of tests

For peer review only

For peer review only - http://bmjopen.bmj.com/site/about/guidelines.xhtml

References

(1) Unal B, Capewell S, Critchley JA. Coronary heart disease policy models: a systematic review. BMC Public Health 2006;6(1):213.

(2) Capewell S, Allender S, Critchley J, Lloyd-Williams F, O'Flaherty M, Rayner M, et al. Modelling the UK burden of cardiovascular disease to 2020: a research report for the Cardio & Vascular Coalition and the British Heart Foundation. British Heart Foundation 2008(Available at http://www.healthimpact.org.uk/content/resources/z154_modelling_the_burden_of_cv_disease.pdf).

(3) Drummond MF, O'Brien B, Stoddart GL, Torrance GW. Methods for the economic evaluation of health care programmes.

(4) Weinstein MC, O'Brien B, Hornberger J, Jackson J, Johannesson M, McCabe C, et al. Principles of good practice for decision analytic modeling in health-care evaluation: report of the ISPOR Task Force on Good Research Practices—Modeling Studies. Value in health 2003;6(1):9-17.

(5) Eddy DM, Hollingworth W, Caro JJ, Tsevat J, McDonald KM, Wong JB, et al. Model transparency and validation: a report of the ISPOR-SMDM Modeling Good Research Practices Task Force-7. Med Decis Making 2012 Sep-Oct;32(5):733-743.

(6) Brennan A, Chick SE, Davies R. A taxonomy of model structures for economic evaluation of health technologies. Health Econ 2006;15(12):1295-1310.

(7) Grover SA, Paquet S, Levinton C, Coupal L, Zowall H. Estimating the benefits of modifying risk factors of cardiovascular disease: a comparison of primary vs secondary prevention. Arch Intern Med 1998;158(6):655-662.

(8) Caro JJ, Huybrechts KF. Stroke treatment economic model (STEM): predicting long-term costs from functional status. Stroke 1999 Dec;30(12):2574-2579.

(9) Van Baal P, Feenstra T, Hoogeveen R, De Wit G. Cost effectiveness analysis with the RIVM Chronic Disease Model. 2005.

(10) Ward S, Jones ML, Pandor A, Holmes M, Ara R, Ryan A, et al. A systematic review and economic evaluation of statins for the prevention of coronary events. 2007.

(11) Smith-Spangler CM, Juusola JL, Enns EA, Owens DK, Garber AM. Population Strategies to Decrease Sodium Intake and the Burden of Cardiovascular DiseaseA Cost-Effectiveness Analysis. Ann Intern Med 2010;152(8):481-487.

(12) Newman J, Grobman WA, Greenland P. Combination Polypharmacy for Cardiovascular Disease Prevention in Men: A Decision Analysis and Cost-Effectiveness Model. Preventive cardiology 2008;11(1):36-41.

(13) Grosso AM, Bodalia PN, MacAllister RJ, Hingorani AD, Moon JC, Scott MA. Comparative clinical- and cost-effectiveness of candesartan and losartan in the management of hypertension and heart failure: a systematic review, meta-and cost-utility analysis. Int J Clin Pract 2011;65(3):253-263.

For peer review only - http://bmjopen.bmj.com/site/about/guidelines.xhtml

For peer review only

(14) Gillespie P, O'Shea E, Murphy AW, Byrne MC, Byrne M, Smith SM, et al. The cost-effectiveness of the SPHERE intervention for the secondary prevention of coronary heart disease. Int J Technol Assess Health Care 2010;26(03):263-271.

(15) Wisloff T, Selmer RM, Halvorsen S, Fretheim A, Norheim OF, Kristiansen IS. Choice of generic antihypertensive drugs for the primary prevention of cardiovascular disease--a cost-effectiveness analysis. BMC Cardiovasc Disord 2012 Apr 4;12:26-2261-12-26.

(16) Nash A, Barry M, Walshe V. Cost effectiveness of statin therapy for the primary prevention of coronary heart disease in Ireland. Ir Med J 2006;99(5):144-145.

(17) Lovibond K, Jowett S, Barton P, Caulfield M, Heneghan C, Hobbs FR, et al. Cost-effectiveness of options for the diagnosis of high blood pressure in primary care: a modelling study. The Lancet 2011;378(9798):1219-1230.

(18) Greving JP, Buskens E, Koffijberg H, Algra A. Cost-effectiveness of aspirin treatment in the primary prevention of cardiovascular disease events in subgroups based on age, gender, and varying cardiovascular risk. Circulation 2008 Jun 3;117(22):2875-2883.

(19) National Institute for Health and Clinical Excellence,. Hypertension: clinical management of primary hypertension in adults. 2011;Clinical Guideline 127.

(20) van Kempen BJ, Ferket BS, Hofman A, Steyerberg EW, Colkesen EB, Boekholdt SM, et al. Validation of a model to investigate the effects of modifying cardiovascular disease (CVD) risk factors on the burden of CVD: the rotterdam ischemic heart disease and stroke computer simulation (RISC) model. BMC Med 2012 Dec 6;10:158-7015-10-158.

(21) Matchar DB, Samsa GP, Matthews JR, Ancukiewicz M, Parmigiani G, Hasselblad V, et al. The Stroke Prevention Policy Model: linking evidence and clinical decisions. Ann Intern Med 1997;127(8_Part_2):704-711.

(22) Lhachimi SK, Nusselder WJ, Smit HA, van Baal P, Baili P, Bennett K, et al. DYNAMO-HIA--a Dynamic Modeling tool for generic Health Impact Assessments. PLoS One 2012;7(5):e33317.

(23) Kang H, Ko S, Liew D. Results of a Markov model analysis to assess the cost-effectiveness of statin therapy for the primary prevention of cardiovascular disease in Korea: the Korean Individual-Microsimulation Model for Cardiovascular Health Interventions. Clin Ther 2009;31(12):2919-2930.

(24) Sorensen SV, Frick KD, Wade A, Simko R, Burge R. Model-based simulation to explore the cost-effectiveness of following practice guidelines for triglyceride and low-density lipoprotein cholesterol control among patients with diabetes mellitus and mixed dyslipidemia. Clin Ther 2009;31(4):862-879.

(25) Whitfield MD, Gillett M, Holmes M, Ogden E. Predicting the impact of population level risk reduction in cardio-vascular disease and stroke on acute hospital admission rates over a 5 year period—a pilot study. Public Health 2006;120(12):1140-1148.

(26) Cooper K, Davies R, Raftery J, Roderick P. Use of a coronary heart disease simulation model to evaluate the costs and effectiveness of drugs for the prevention of heart disease. J Oper Res Soc 2008;59(9):1173-1181.

For peer review only - http://bmjopen.bmj.com/site/about/guidelines.xhtml

(27) Babad H, Sanderson C, Naidoo B, White I, Wang D. The development of a simulation model of primary prevention strategies for coronary heart disease. Health Care Manag Sci 2002;5(4):269-274.

(28) Gunning-Schepers L, Barendregt J, van der Maas P. PREVENT, a Model to Estimate the Health Benefits of Prevention. Models of Non-communicable Diseases.Health status and Health Service Requirements 1992:55-69.

(29) Keaver L, Webber L, Dee A, Shiely F, Marsh T, Balanda K, et al. Application of the UK foresight obesity model in Ireland: the health and economic consequences of projected obesity trends in Ireland. 2013.

(30) Manuel DG, Tuna M, Hennessy D, Bennett C, Okhmatovskaia A, Fines P, et al. Projections of preventable risks for cardiovascular disease in Canada to 2021: a microsimulation modelling approach. CMAJ Open 2014 May 20;2(2):E94-E101.

(31) Annemans L, Lamotte M, Clarys P, Van den Abeele E. Health economic evaluation of controlled and maintained physical exercise in the prevention of cardiovascular and other prosperity diseases. Eur J Cardiovasc Prev Rehabil 2007 Dec;14(6):815-824.

(32) Cecchini M, Sassi F, Lauer JA, Lee YY, Guajardo-Barron V, Chisholm D. Tackling of unhealthy diets, physical inactivity, and obesity: health effects and cost-effectiveness. The Lancet 2010;376(9754):1775-1784.

(33) Ara R, Blake L, Gray L, Hernandez M, Crowther M, Dunkley A, et al. What is the clinical effectiveness and cost-effectiveness of using drugs in treating obese patients in primary care? A systematic review. 2012.

(34) Department of Health. Economic Modelling for Vascular Checks. 2008; Available at: www.healthcheck.nhs.uk/document.php?o=225. Accessed July, 2015.

(35) Green N, Smith D, Sperrin M, Buchan I. A Novel Chronic Disease Policy Model. arXiv preprint arXiv:1009.0405 2010.

(36) Weinstein MC, Coxson PG, Williams LW, Pass TM, Stason WB, Goldman L. Forecasting coronary heart disease incidence, mortality, and cost: the Coronary Heart Disease Policy Model. Am J Public Health 1987 Nov;77(11):1417-1426.

(37) Fidan D, Unal B, Critchley J, Capewell S. Economic analysis of treatments reducing coronary heart disease mortality in England and Wales, 2000-2010. QJM 2007 May;100(5):277-289.

(38) Sundberg G, Bagust A, Terént A. A model for costs of stroke services. Health Policy 2003;63(1):81-94.

(39) Mihalopoulos C, Cadilhac DA, Moodie ML, Dewey HM, Thrift AG, Donnan GA, et al. Development and application of Model of Resource Utilization, Costs, and Outcomes for Stroke (MORUCOS): An Australian economic model for stroke. Int J Technol Assess Health Care 2005;21(04):499-505.

(40) Lauer JA, Rohrich K, Wirth H, Charette C, Gribble S, Murray CJ. PopMod: a longitudinal population model with two interacting disease states. Cost Eff Resour Alloc 2003 Feb 26;1(1):6.

For peer review only - http://bmjopen.bmj.com/site/about/guidelines.xhtml

(41) Tobias M, Sexton K, Mann S, Sharpe N. How low can it go? Projecting ischaemic heart disease mortality in New Zealand to 2015. N Z Med J 2006;119(1232).

(42) Murray CJ, Lopez AD. Alternative projections of mortality and disability by cause 1990–2020: Global Burden of Disease Study. The Lancet 1997;349(9064):1498-1504.

(43) Struijs JN, van Genugten ML, Evers SM, Ament AJ, Baan CA, van den Bos, Geertrudis AM. Modeling the future burden of stroke in the Netherlands impact of aging, smoking, and hypertension. Stroke 2005;36(8):1648-1655.

(44) Malik IS, Bhatia VK, Kooner JS. Cost effectiveness of ramipril treatment for cardiovascular risk reduction. Heart 2001 May;85(5):539-543.

(45) Schau B, Boysen G, Truelsen T, Boden-Albala B, Cheng J, Babamoto E, et al. Development and validation of a model to estimate stroke incidence in a population. Journal of Stroke and Cerebrovascular Diseases 2003;12(1):22-28.

(46) Schlessinger L, Eddy DM. Archimedes: a new model for simulating health care systems—the mathematical formulation. J Biomed Inform 2002;35(1):37-50.

(47) National Institute for Health and Care Excellence (NICE). Lipid modification: cardiovascular risk assessment and the modification of blood lipids for the primary and secondary prevention of cardiovascular disease. 2014; Clinical Guideline 181.

For peer review only
